# Three Gorges Dam: friend or foe of riverine greenhouse gases?

**DOI:** 10.1093/nsr/nwac013

**Published:** 2022-01-28

**Authors:** Jinren Ni, Haizhen Wang, Tao Ma, Rong Huang, Philippe Ciais, Zhe Li, Yao Yue, Jinfeng Chen, Bin Li, Yuchun Wang, Maosheng Zheng, Ting Wang, Alistair G L Borthwick

**Affiliations:** Key Laboratory for Water and Sediment Sciences, Ministry of Education, College of Environmental Sciences and Engineering, Peking University, Beijing 100871, China; State Environmental Protection Key Laboratory of All Materials Fluxes in River Ecosystems, Ministry of Ecology and Environment, Beijing 100871, China; Key Laboratory for Water and Sediment Sciences, Ministry of Education, College of Environmental Sciences and Engineering, Peking University, Beijing 100871, China; Key Laboratory for Water and Sediment Sciences, Ministry of Education, College of Environmental Sciences and Engineering, Peking University, Beijing 100871, China; Key Laboratory for Water and Sediment Sciences, Ministry of Education, College of Environmental Sciences and Engineering, Peking University, Beijing 100871, China; Laboratoire des Sciences du Climat et de l’Environnement, Institut Pierre Simon Laplace, Commissariat à l’Énergie Atomique et aux Ènergies Alternatives, CNRS, Université de Versailles Saint-Quentin-en-Yvelines, Gif-sur-Yvette 91191, France; Key Laboratory of Reservoir Environment, Chongqing Institute of Green and Intelligent Technology, Chinese Academy of Sciences, Chongqing 400714, China; State Key Laboratory of Water Resources and Hydropower Engineering Science, Wuhan University, Wuhan 430072, China; Key Laboratory for Water and Sediment Sciences, Ministry of Education, College of Environmental Sciences and Engineering, Peking University, Beijing 100871, China; Key Laboratory for Water and Sediment Sciences, Ministry of Education, College of Environmental Sciences and Engineering, Peking University, Beijing 100871, China; Department of Water Environment, China Institute of Water Resources and Hydropower Research, Beijing 100038, China; MOE Key Laboratory of Regional Energy Systems Optimization, Resources and Environmental Research Academy, North China Electric Power University, Beijing 102206, China; Key Laboratory for Water and Sediment Sciences, Ministry of Education, College of Environmental Sciences and Engineering, Peking University, Beijing 100871, China; School of Engineering, The University of Edinburgh, Edinburgh EH9 3JL, UK

**Keywords:** Three Gorges Dam, greenhouse gas, spatiotemporal variation, equilibrium, Yangtze River, whole system analysis

## Abstract

Dams are often regarded as greenhouse gas (GHG) emitters. However, our study indicated that the world's largest dam, the Three Gorges Dam (TGD), has caused significant drops in annual average emissions of CO_2_, CH_4_ and N_2_O over 4300 km along the Yangtze River, accompanied by remarkable reductions in the annual export of CO_2_ (79%), CH_4_ (50%) and N_2_O (9%) to the sea. Since the commencement of its operation in 2003, the TGD has altered the carbonate equilibrium in the reservoir area, enhanced methanogenesis in the upstream, and restrained methanogenesis and denitrification via modifying anoxic habitats through long-distance scouring in the downstream. These findings suggest that ‘large-dam effects’ are far beyond our previous understanding spatiotemporally, which highlights the fundamental importance of whole-system budgeting of GHGs under the profound impacts of huge dams.

## Teaser

Whole system thinking subverted the conventional perception that dams would enhance GHGs emission in a large river.

## INTRODUCTION

Most rivers worldwide are supersaturated with greenhouse gases (GHGs) owing to inputs of carbon (C) and nitrogen (N) from land, and become net sources of GHGs for the atmosphere [1]. To meet the growing global demand for water and energy, more than 70 000 large dams have been constructed [2]. Such dams are regarded as a source of excessive GHG emissions [3–5]. The estimated annual emissions are 48 Tg C as CO_2_ and 3 Tg C as CH_4_ from global hydropower reservoirs, and 0.03 Tg N as N_2_O from all reservoirs in the world [4,6].

Previous studies on the effects of dams on GHGs have been mostly limited to the vicinity of reservoirs [7–10]. Although these considerations hold for small dams (reservoir capacity < 10 km^3^), the impact of large dams on GHGs (reservoir capacity ≥ 10 km^3^) is much greater because the original physical and biochemical equilibria are disrupted over large spatiotemporal scales. Firstly, a large dam alters the hydrodynamic conditions and material fluxes of a river: after operation commences, the peak flood discharge decreases and fluxes of nutrients and sediments exported to the sea are often reduced [11–14]. Secondly, the river regime tends to remain stable, but increasing longitudinal erosion of the riverbed beyond the dam causes long-term readjustment over a considerable distance [15]. Thirdly, changes to water and sediment fluxes significantly affect the functioning of microbial communities [16–18] (e.g. photosynthesis, methanogenesis and denitrification) and GHG emissions (Supplementary Table 1).

As the world's largest dam, the Three Gorges Dam (TGD) has been regarded as a significant source of GHG emissions [3,4,19]. For example, CO_2_ and CH_4_ emissions from the 25 km^2^ core reservoir area upstream of the TGD in 2008 were estimated to be 40 and 20 Gg yr^–1^, respectively, ∼40- and 20-fold larger than before impoundment [20]. Similar findings [4,21] reported that the total CH_4_ emission rate in the Three Gorges Reservoir (TGR) was 0.315 Gg yr^–1^. However, the impact of the TGD extends far beyond the reservoir area. The TGD has altered hydrodynamic conditions along almost the entire length of the Yangtze, as physical and biochemical processes have readjusted both upstream and downstream of the dam, most notably the long-distance, long-term scouring of the riverbed downstream of the dam [15,22,23]. This highlights the necessity of whole-river analysis in order to properly assess the changes in GHG fluxes caused by large dams.

**Figure 1.**
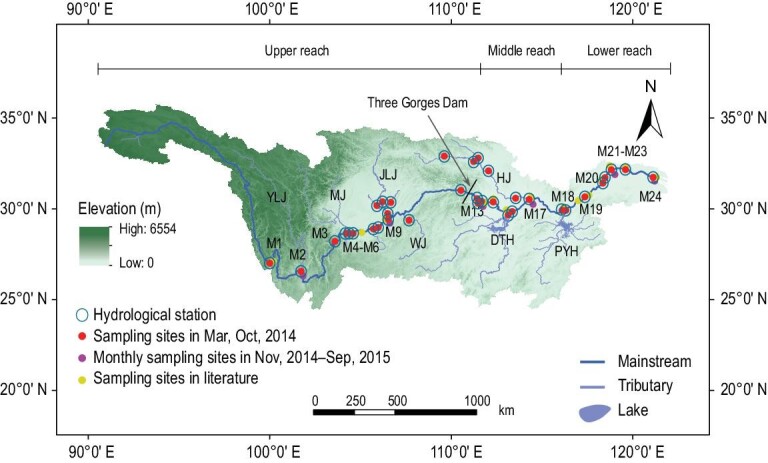
The Yangtze River Basin and sampling sites. Lines indicate the mainstream river and its tributaries, the former having a length of 4300 km (i.e. the actual sinuous channel length, equivalent to 2.05 times the straight-line distance of 2102 km from the start to the end of the sampling sites). Yellow solid circles show the locations of previous sampling sites (see Supplementary Tables 2–3); red solid circles show the locations of our recent simultaneous sampling sites in March and October 2014 (for details see Supplementary Table 4); purple solid circles show the locations of our monthly sampling sites from October 2014 to September 2015; blue open circles show the locations of the hydrological stations. The upper reach is from Shigu (M1) to Yichang (M13), the middle reach from Yichang to Hukou (M18) and the lower reach from Hukou to Xuliujing (M24). The major tributaries include Yalongjiang (YLJ), Minjiang (MJ), Jialingjiang (JLJ), Wujiang (WJ) and Hanjiang (HJ); two river-regulated lakes are Dongting (DTH) and Poyang (PYH).

Here, we estimate changes in dissolved and emitted fluxes of GHGs in the Yangtze River before and after the TGD became operational in 2003. Based on the time series of 30 water quality indices monitored over 312 months (1990–2015) and the measured GHGs (Supplementary Tables 2–4) along 4300 km of the Yangtze River (Fig. 1), CO_2_ is calculated using the well-known CO2SYS model, while CH_4_ and N_2_O are estimated with artificial neural networks (ANNs; see Methods).

## RESULTS AND DISCUSSION

### Temporal effect of the TGD on CO_2_ fluxes

**Figure 2.**
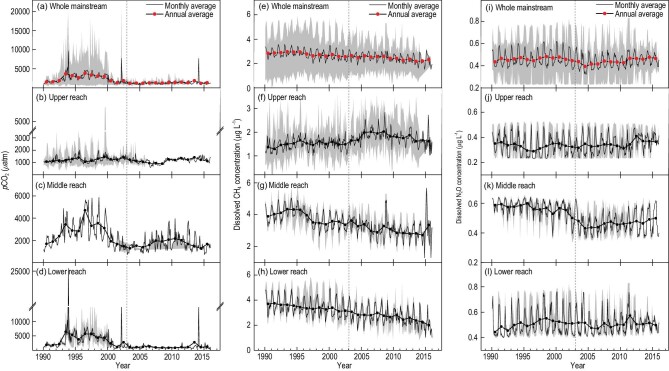
Temporal variations in monthly and annual averages of dissolved-GHG concentrations from 1990 to 2015: (a–d) *p*CO_2_, (e–h) dissolved CH_4_, and (i–l) dissolved N_2_O. The shadow areas represent the range of dissolved-GHG concentrations at different monitoring stations in the corresponding reaches. Vertical dashed lines denote 2003, when the TGD commenced operation.

The mean annual *p*CO_2_ between 1990 and 2002 was 2526 μatm (Fig. 2). Subsequently, *p*CO_2_ declined greatly to 1336 μatm once the TGD began operation over the whole mainstream (Fig. 2a). This declining trend is particularly significant in the middle and lower reaches, though annual *p*CO_2_ in the upper reach remained relatively steady before and after 2003 (Fig. 2b–d). The spatially averaged annual *p*CO_2_ was ${\rm{2205}}_{\ {\rm{ - 925}}}^{{\rm{\ + 2497}}}$ μatm (where the numbers display the mean and range of values) in the middle reach. *p*CO_2_ increased to 2974 μatm during the 1990s, peaked in 1996 and declined significantly to 1720 μatm after TGD impoundment [24] (Fig. 2c). In the middle reach, *p*CO_2_ decreased from 2907 to 1446 μatm in the wet season and from 2196 to 1377 μatm in the dry season (Supplementary Fig. 1a–d).

From 1990 to 2015, CO_2_ exported to the East China Sea exhibited substantial inter-annual variations (Supplementary Fig. 2). The mean annual value increased from ∼469 Gg C yr^–1^ in 1993 and reached a peak of 3354 Gg C yr^–1^ during the 1998 flood before declining to pre-1993 levels by 2003 (Supplementary Fig. 2). The mean exported CO_2_ flux from 1991 to 2015 was 1128 Gg C yr^–1^, corresponding to 5.6% of dissolved inorganic carbon transported by the Yangtze River (Supplementary Table 5). The annual averaged CO_2_ outgassing flux and CO_2_ exported to the sea over the Yangtze experienced remarkable drops of 55% and 79% since 2003, suggesting a much stronger effect, due to TGD impoundment, on *p*CO_2_ than that from other influencing factors (such as the anthropogenic discharge of sulfur and nitrogen containing pollutants) reported previously [24].

Monthly and annual CO_2_ emission fluxes from the upper, middle and lower reaches were on average lower after 2003 than before, indicating that the entire mainstream progressively became a smaller emission source (Supplementary Fig. 3). The largest change occurred in the middle and lower reaches, where CO_2_ emission flux dropped from 2723 Gg C yr^–1^ before TGR impoundment to 1087 Gg C yr^–1^ after. Annual averaged CO_2_ emission flux from the Yangtze mainstream was estimated as ${\rm{2420}}_{\ {\rm{ - 1200}}}^{{\rm{\ + 2590}}}\ $Gg C yr^–1^ (Supplementary Table 6), which accounts for emissions from 1.3% of global rivers and 4.8% of temperate rivers [1,25] between 25°N and 50°N. These results were convincing with uncertainty analysis based on representative stations as described in the Supplementary Data.

### Temporal effect of the TGD on CH_4_ fluxes

To estimate dissolved and emitted CH_4_ over the Yangtze River before and after impoundment of the TGR, monthly observed data of chemical oxygen demand, dissolved oxygen, water temperature, pH and nitrogen during 1990–2015 were used for validation and verification as input variables of ANN models (see Methods). Supplementary Fig. 4 shows spatiotemporal variations in dissolved nitrogen (NH_4_^+^, NO_3_^–^, NO_2_^–^) in the whole mainstream during the period 1990–2015.

After the TGR impoundment in 2003, both dissolved and emitted CH_4_ concentrations increased in the upper reach, decreased in the middle reach and hardly changed in the lower reach (Fig. 2f–h, Supplementary Fig. 5b–d). The annual averaged CH_4_ concentration from 1990 to 2015 over the whole mainstream was ${\rm{2}}{\rm{.22}}_{\ {\rm{ - 0}}{\rm{.65}}}^{{\rm{\ + 0}}{\rm{.54}}}$ μg L^–1^ (Fig. 2e), comparable to that for the Amazon River (Supplementary Table 7) [26]. The mean dissolved CH_4_ was ${\rm{3}}{\rm{.15}}_{\ {\rm{ - 0}}{\rm{.56}}}^{{\rm{\ + 0}}{\rm{.62}}}\ $μg L^–1^ in the dry season and ${\rm{2}}{\rm{.57}}_{\ {\rm{ - 0}}{\rm{.72}}}^{{\rm{\ + 0}}{\rm{.59}}}\ $μg L^–1^ in the wet season in the Yangtze (Supplementary Fig. 1). A major change in seasonal cycles of dissolved CH_4_ occurred in 2003. In the wet season, the mean dissolved CH_4_ increased from 1.45 to 1.95 μg L^–1^ in the upper reach but decreased from 3.51 to 3.02 μg L^–1^ in the middle reach. Based on the parameters derived from representative stations (Supplementary Table 8), temporal variation in CH_4_ flux exported to the East China Sea decreased from 3.1 to 1.5 Gg C yr^–1^ after 2003 (Supplementary Fig. 6). Emitted CH_4_ flux decreased from 3.3 to 2.7 Gg C yr^–1^ along the whole mainstream, having increased from 0.4 to 0.5 Gg C yr^–1^ upstream of the dam and decreased from 2.9 to 2.2 Gg C yr^–1^ downstream of the dam since the operation of the TGD (Supplementary Fig. 7).

### Temporal effect of the TGD on N_2_O fluxes

**Figure 3. fig3:**
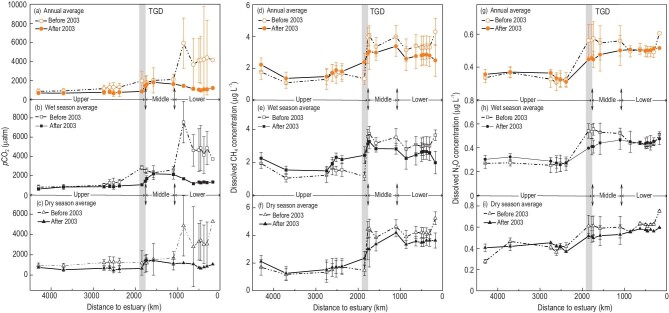
Spatial variations in annual and seasonal dissolved-GHG concentrations in the 4300-km stretch of the Yangtze River: (a–c) dissolved-CO_2_ concentration, (d–f) dissolved CH_4_ concentration, and (g–i) dissolved N_2_O concentration. The error bars are the annual standard deviations at the given monitoring stations. The shaded area indicates where the TGD reservoir is located.

Input variables in the ANN model for estimation of N_2_O emissions included dissolved oxygen, water temperature, pH and nitrogen. Total dissolved nitrogen[Fig fig3] (NH_4_^+^ + NO_3_^–^ + NO_2_^–^) increased during the period of interest, while NH_4_^+^ and NO_2_^–^ had much lower concentration levels than NO_3_^–^ (Supplementary Fig. 4). This is consistent with increasing nitrogen input from fertilizers to the Yangtze River basin in the past few decades, enhanced by population and economic growth in central and east China [27,28]. After training and verification of the ANN, the modeled results showed a slight reduction of dissolved and emitted N_2_O owing to the dam’s operation since 2003. Over the Yangtze mainstream, the annual average concentration was ${\rm{0}}{\rm{.45}}_{\ {\rm{ - 0}}{\rm{.22}}}^{{\rm{\ + 0}}{\rm{.38}}}$ μg L^–1^ (Fig. 2i), demonstrating a moderate dissolved N_2_O concentration compared with other large rivers (Supplementary Table 9). Dissolved N_2_O reached a maximum of 0.55 μg L^–1^ at the Xuliujing station in the river mouth (Fig. 2l), and a minimum of 0.32 μg L^–1^ at the Luzhou station in the upper reach (Fig. 2j). Impoundment of the TGR operation caused dissolved N_2_O to decrease from 0.56 to 0.46 μg L^–1^ in the middle reach after 2003 (Fig. 2k). Large amplitude variations in seasonal N_2_O patterns also occurred in the middle reach (Supplementary Fig. 1k). After 2003, the average dissolved N_2_O concentration declined from 0.61 to 0.51 μg L^–1^ in the dry season and from 0.54 to 0.41 μg L^–1^ in the wet season in the middle reach. Seasonal differences of N_2_O emission rates were also calculated (Supplementary Fig. 8e–h). The long-term average (1990–2015) displayed higher N_2_O emission rates at Yichang and Wuhan in the wet season than in the dry season, in all cases indicating the Yangtze was a net source of N_2_O (Supplementary Fig. 8). Meanwhile, N_2_O emission rates at Yichang have fallen from 39.3 to 19.2 μg m^–2^ h^–1^ during the wet season and from 18.4 to 11.6 μg m^–2^ h^–1^ during the dry season (Supplementary Fig. 8g). Based on monthly dissolved N_2_O and flow discharge, the highest values of N_2_O fluxes to the estuary occurred in 1998, the year with historical floods. Mean annual dissolved N_2_O fluxes to the estuary decreased from 0.46 to 0.41 Gg N yr^–1^ after TGD impoundment in 2003 (Supplementary Fig. 9), because of the disruptive effect on the physical and biochemical equilibria of the river. The annual N_2_O outgassing in the mainstream was estimated as 0.43 Gg N yr^–1^ (Supplementary Fig. 10).

### Spatial effect of the TGD on GHG emissions

**Figure 4. fig4:**
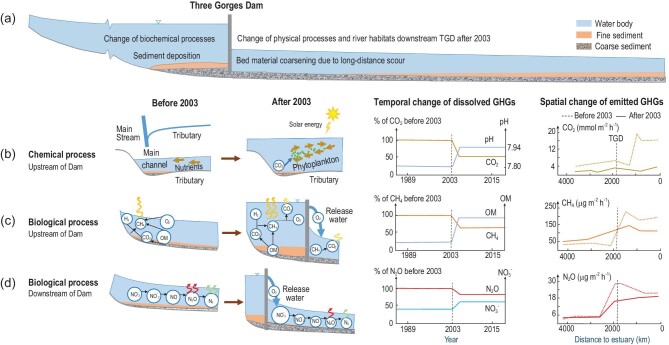
(a–d) Whole-system analysis concerning readjustment of physical and biogeochemical equilibria involved in the regulation effects of the TGD on GHG emissions from the Yangtze River.

Before 2003, *p*CO_2_ ranged from 880 to 4399 μatm in the mainstream channel of the Yangtze River (Fig. 3a).[Fig fig4] A trend of increasing *p*CO_2_ was evident along the mainstream, rising from 1314 μatm in the upper reach to 4111 μatm in the lower reach, along with the decreasing pH level of the lower reach and dilution by water entering from Poyang Lake during the period 1990–2002. After 2003, *p*CO_2_ was almost constant upstream of the TGD, but rose immediately downstream of the dam, affected by flow regulation and sediment trapping [29]. It has been estimated that reservoir sedimentation caused by the presence of a dam results in an average carbon accumulation rate of 400 g m^–2^ yr^–1^ globally [30]. Carbon burial therefore results in a potential available carbon source for biological respiration and might increase *p*CO_2_ in a reservoir, particularly in the early years after impoundment [31]. Other human activities might also increase exchanges between water and mineral, thus increasing *p*CO_2_ [32]. Similar trends of increasing *p*CO_2_ were observed along the mainstream in both wet and dry seasons (Fig. 3b–c). The higher values of *p*CO_2_ in the wet season compared to the dry season, especially in middle and lower reaches, might be due to the efficient production of soil-originated CO_2_ and its transportation by surface run-off [31]. Supplementary Fig. 11 shows the CO_2_ emission rate profiles along the mainstream before and after operation of the TGD. These are qualitatively very similar to the dissolved CO_2_ profiles. After 2003, the mean CO_2_ emission rate along the mainstream was 3.0 ± 1.7 mmol m^–2^ h^–1^. Degassing rates were higher in the middle and lower reaches than in the upper reach, controlled by *p*CO_2_.

CH_4_ concentration was lowest in the upper reach of the Yangtze in both wet and dry seasons (Fig. 3d–f), primarily because of lower levels of organic matter. After 2003, CH_4_ concentration increased slightly from 1.50 to 1.83 μg L^–1^ in the upper reach, and decreased from 3.13 to 2.74 μg L^–1^ in the lower reach (Fig. 3d). The TGD impoundment influenced the CH_4_ emission rate in a trend similar to that of its dissolved concentration (see Supplementary Fig. 5).

The TGD influenced N_2_O distributions both upstream and downstream of the dam, especially in the middle reach of the Yangtze (Fig. 3g). After 2003, annual averaged N_2_O concentrations decreased slightly from 0.42 to 0.38 μg L^–1^ in the wet season and from 0.55 to 0.50 μg L^–1^ in the dry season (Fig. 3h–i). The most remarkable decrease in N_2_O concentration occurred at Yichang, immediately downstream of the TGD (Supplementary Fig. 12a). At Yichang, monthly averaged N_2_O emission rates fell both in the wet and dry seasons, and the amplitude of the fluctuations in N_2_O emission rate also declined (Supplementary Fig. 12a) with smaller seasonal differences (Supplementary Fig. 12b) after TGD impoundment.

### GHG fluxes in response to readjustment of physical and biochemical equilibria

Our study indicated that the TGD has caused significant drops in the overall annual GHG fluxes emitted to the atmosphere and exported to the sea since 2003 (Supplementary Table 10). To interpret such changes, a whole-river analysis (Fig. 4) must be made of the readjustments to hydrodynamic conditions (Fig. 4a) and biogeochemical equilibria (Fig. 4b–d) over the broader spatiotemporal scale of the river.

#### Cause of CO_2_ drop

Due to TGD impoundment, a backwater zone developed upstream of the dam wherein water exchanges took place between the mainstream and tributaries (Fig. 4b). Water retention time significantly increased in the reservoir in addition to the significantly decreased flow velocity (<0.2 m s^–1^) in some tributaries entering into the reservoir. Such changes replenish nutrients in the tributaries via circulation with the mainstream [33]. Accumulated nutrients and restricted vertical mixing in the backwater area of the tributaries favored phytoplankton growth [34,35], causing algae to flourish [36] (Supplementary Table 11). Algae’s photosynthetic removal of CO_2_ and bioaccumulation of NO_3_^–^, H_2_PO_4_^–^, HPO_4_^2–^ and PO_4_^3–^ resulted in a higher pH in the tributaries, promoting acceleration of eutrophication [37,38]. The higher pH in the tributaries helped neutralize hydrogen ions in the mainstream, breaking the carbonate equilibrium of the river and ultimately leading to a sharp drop in CO_2_ in the mainstream (Supplementary Fig. 13).

#### Cause of CH_4_ drop

Although CH_4_ increased upstream, a net reduction of CH_4_ emissions (∼17%) happened along the whole mainstream after the TGR impoundment, due to a decrease in CH_4_ downstream of the TGD. The input of dissolved CH_4_ into the ocean decreased by 50%, primarily because the TGD modified the GHG regime and disrupted the biotic equilibrium of the Yangtze (Fig. 4c). Upstream of the TGD, both dissolved and emitted CH_4_ increased after the reservoir impoundment, owing to the effects of flow regulation and sediment trapping. Such carbon burial promotes heterotrophic methanogenesis, thus increasing the dissolved CH_4_ content of the reservoir [29]. Anoxic conditions due to increased water depth in front of the dam would also be beneficial to methanogens locally [11]. However, both dissolved and emitted CH_4_ declined downstream of the dam, mainly because of riverbed scouring, which damaged the habitat of anaerobic *Archaea* responsible for heterotrophic methanogenesis [39,40]. In addition, the pre-impoundment clearance also reduced decomposition of organic carbon and inhibited the significant increase in CH_4_ emissions in the TGR. During reservoir flushing, degassing would occur because of rapid depressurization and strong aeration, resulting in increased emissions of dissolved CH_4_ and lowering of CH_4_ concentration downstream [6,41]. Overall, the TGD regulated the CH_4_ emission regime of the Yangtze, causing dissolved CH_4_ to increase in the upper reach and decrease in the lower reach.

#### Cause of N_2_O drop

N_2_O flux emissions over the mainstream decreased from 0.44 to 0.41 Gg N yr^–1^, and N_2_O exports to the sea fell from 0.46 to 0.41 Gg N yr^–1^ after TGD operation commenced. Land use changes and water quality protection measures resulted in low nitrogen loading to the TGR. Formation of hypoxia or even anoxia in the reservoir was generally restricted (Fig. 4d). The promoted denitrification, whereby N_2_O was transformed directly to N_2_, caused N_2_O to decrease slightly upstream of the dam [42–44]. On the other hand, riverbed scouring downstream of the TGD altered the habitat of heterotrophic denitrifiers, slowing down denitrification. This is consistent with our findings of high NO_3_^–^ concentration but low NO_2_^–^ concentration in the river [45] (Supplementary Figs 4 and 14a–b). Again, reservoir flushing would have raised degassing of N_2_O and N_2_. Discharge of cooler, high-pressure bottom water, supersaturated with gases, from the 175-m-deep reservoir to the warmer, low-pressure downstream river would enhance N_2_O emissions [14]. Riverine microbial communities require phosphorus as a nutrient, and pH to regulate nitrification and denitrification processes. The estimated annual mass of reactive phosphorus retained by dams along the Yangtze was 0.5 Gmol yr^–1^ in 2010, and it will rise to 2.9 Gmol yr^–1^ by 2030; this would alter denitrification, thus decreasing N_2_O production. Hence, the influence of phosphorus is likely to be significantly less than riverbed scouring on the nitrogen cycle downstream of the TGD. Field observations also exhibited an increase in pH downstream of the TGD since 2003; this encouraged nitrification, as evidenced by the very low levels of ammonium that were recorded (Supplementary Fig. 14).

**Figure 5.**
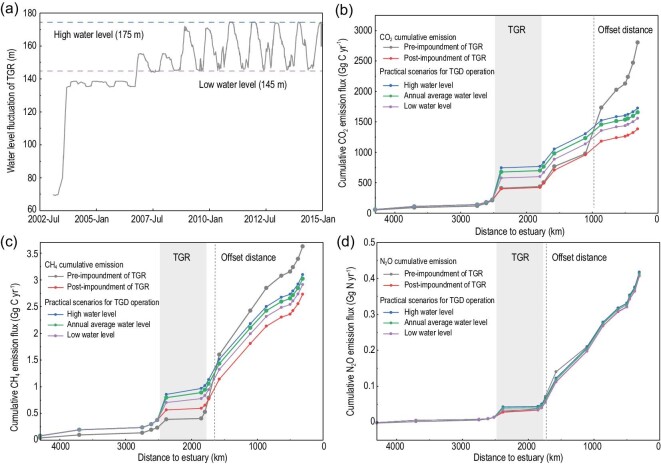
The balance of GHG emission fluxes enlarged by the reservoir itself and those reduced by habitat modification downstream from the dam under practical TGD operation. According to (a) different scenarios for the annual variation of the TGD’s operating water level, the offset distance was (b) 766–819 km for CO_2_, (c) 124–180 km for CH_4_ and (d) 18–53 km for N_2_O downstream from the dam, respectively. Under the averaged operating water level, the vertical dotted lines indicate the locations where the changed GHG emission fluxes, due to the reservoir, were offset by the decreased GHG emissions in the downstream of the dam.

Lastly, the key concern becomes how the enlargement of CO_2_ (1.8 × 10^2^–3.4 × 10^2^ Gg C yr^–1^), CH_4_ (0.18–0.37 Gg C yr^–1^) and N_2_O (0.0072–0.01 Gg N yr^–1^) emissions caused by the reservoir itself would be finally offset by the reduction of GHG emissions resulting from downstream habitat modification. According to pre-impoundment estimates of GHG fluxes from the reservoir and post-impoundment measurements on possible GHG pathways, such a balancing out would be expected at 766–819 km (for CO_2_), 124–180 km (for CH_4_) and 18–53 km (for N_2_O) downstream of the TGD, respectively (Fig. 5). Under the practical scenarios for TGD operation [46] (Supplementary Table 12), the overall net reduction in GHG emissions would still be significant (38.43%–44.60% for CO_2_, 14.51%–19.70% for CH_4_ and 0.21%–2.50% for N_2_O) in the entire Yangtze. In the reservoir area, the river-valley geomorphology restricted the rise of the littoral shallow area (<10 m), resulting in less CH_4_ and CO_2_ emissions from ebullition (<8% in the gross GHG emission estimates of the TGR, see Supplementary Table 13). Sensitivity analysis confirmed the availability of the study results under uncertainties from the models and those induced by the TGR (Supplementary Figs 15 and 16). In the balance, the net change in GHG emissions directly caused by the TGR could alter neither the dominant GHG emission pathways from the reservoir nor the general GHG reduction trend from the perspective of the full 4300 km along the mainstream of the Yangtze River (for details see Section 9 in the Supplementary Data).

## CONCLUSIONS

In contrast to the general claim that dams increase emissions of GHGs from rivers, we found that the TGD, the world's largest dam, caused a significant reduction in annual average emissions of CO_2_, CH_4_ and N_2_O over a 4300-km stretch of the Yangtze River. Meanwhile, a remarkable drop occurred in the annual export of CO_2_ (79%), CH_4_ (50%) and N_2_O (9%) to the sea from the river. These findings suggest that more profound impacts are produced by the ‘large dams’ than are expected from ‘small dams’, whose effects are limited to the vicinity of reservoirs, either spatially or temporally. The impoundment of a large reservoir not only altered the environment in the reservoir area, but also resulted in significant changes to riverine habitats downstream. In particular, long-term and long-distance riverbed erosion downstream of the large dam essentially changes the processes of photosynthesis, methanogenesis and denitrification, commencing the re-establishment of the biogeochemical equilibrium over the whole river system. This highlights the primary importance of whole-system analysis in understanding the complex effects of large dams on readjustments of physical, chemical and biological equilibria in large rivers globally.

## METHODS

Water quality was monitored monthly at 43 hydrological stations (blue open circles, Fig. 1). Simultaneous sampling of hydrological, environmental and all GHG constituents was undertaken in the spring and autumn of 2014 along the 4300-km stretch (i.e. the actual sinuous channel length, equivalent to 2.05 times the straight-line distance of 2102 km from the start to the end of the sampling sites; red circles, Fig. 1). Further monthly sampling took place from November 2014 to September 2015 at six stations (purple solid circles, Fig. 1). Given the limited data available for model establishment (Supplementary Tables 2–3), we included data from previous studies conducted at certain sites along the Yangtze River. Details of model verification are given in Supplementary Tables 14 and 15. All samples were collected in triplicate. Dissolved CO_2_, CH_4_ and N_2_O were determined using the headspace equilibration technique [47]. CO_2_, CH_4_ and N_2_O emission rates were measured using the static floating chamber technique [47,48]. CO_2_, CH_4_ and N_2_O concentrations were obtained using a gas chromatograph.

Water chemistry monitoring was conducted by the Changjiang Water Resources Commission on a monthly basis from 1990 to 2015. pH, total alkalinity, HCO_3_^–^, water temperature (T), *p*CO_2_ and dissolved CO_2_ concentrations were determined at 18 stations (Supplementary Table 16). As described in Supplementary Figs 17 and 18, ANNs based on backward propagation were used to calculate dissolved CH_4_ (with inputs of chemical oxygen demand, dissolved oxygen, water temperature, pH, NO_3_^–^ and NH_4_^+^) and N_2_O (with inputs of NH_4_^+^, NO_2_^–^, NO_3_^–^, dissolved oxygen, water temperature and pH). The model validation of dissolved CH_4_ and N_2_O concentrations (including data from previous studies conducted at certain sites along the Yangtze River) is shown in Supplementary Figs 19 and 20. Sensitivity analysis was performed by changing input variables (Supplementary Figs 15 and 16). For comparison, calculated dissolved N_2_O concentrations from previous regression models are listed in Supplementary Table 17. The GHG emission rate across the air–water interface was calculated using a two-layer diffusive gas exchange model [49]. Herein, *k*_600_ is an important parameter for calculating the gas emission rate from the dissolved gas concentration. Based on the re-examination of existing empirical formulas for *k*_600_ (Supplementary Table 18), *k*_600_ was determined for the monitoring sites at different reaches of the Yangtze River (Supplementary Table 19). Wind speed data near the hydrological stations were extracted from the China Meteorological Data Sharing Service System (http://data.cma.gov.cn). The atmospheric CH_4_ concentration was assumed to be equivalent to the monthly averaged global background concentration at six monitoring stations across the world (NOAA/CMDL/CCGG air sampling network, http://www.cmdl.noaa.gov/). Model validation and parameter (e.g. *k*_600_) determination are detailed in the Supplementary Data.

## Supplementary Material

nwac013_Supplemental_Files
