# Supplementary material for: Three Gorges Dam: friend or foe of riverine greenhouse gases?
Source: Natl Sci Rev. 2022 Jan 28;9(6):nwac013. doi: 10.1093/nsr/nwac013 (PMC9166553; doi:10.1093/nsr/nwac013)
Supplement: nwac013_Supplemental_Files [file nwac013_supplemental_files.zip › SI_NSR-Ni.docx]

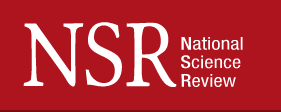


Supplementary Data for

**Three Gorges Dam: Friend or Foe of Riverine Greenhouse Gases?**

Jinren Ni^*^, Haizhen Wang, Tao Ma, Rong Huang, Philippe Ciais, Zhe Li, Yao Yue, Jinfeng Chen, Bin Li, Yuchun Wang, Maosheng Zheng, Ting Wang, Alistair G.L. Borthwick

Correspondence to jinrenni@pku.edu.cn

**This PDF file includes:**

Supplementary Text (page 1~16)

Figs. S1 to S22 (page 17~38)

Tables S1 to S19 (page 39~57)

References (page 58~65)

**Supplementary Text**

# 1. The Yangtze River basin

The Yangtze River is the longest river in Asia. The river has an average annual runoff of 9.3×10^11^ m^3^, drains an area of 1.8×10^6^ km^2^ of central China and flows eastwards through 11 provinces, receiving water from several major tributaries such as the Yalong, Min, Jialing, Wu, and Han rivers and exchanging water with the two largest lakes (i.e. the Poyang Lake and the Dongting Lake) in China [50]. Characterized by a multistage ladder terrain, the river flows from mountainous and plateau regions (Qinghai-Tibet Plateau) through hill and basin regions (Sichuan Basin) to the Middle-Lower Yangtze Plain before entering the East China Sea (Fig. 1). The Yangtze River basin experiences a typical monsoon climate, with a wet season from July to September and a dry season from January to March each year. The highest temperature usually occurs in July and the lowest in January. Precipitation in the river basin is unevenly distributed, with mean annual rainfall of 1,100 mm.

Over the past decades, numerous dams have been constructed in the river basin, and the world’s largest dam, the Three Gorges Dam (TGD), is located near Yichang at the junction between the upper and middle reaches of the Yangtze (Fig. 1). Since operation commenced in 2003, the TGD has met multiple objectives including flood control, ship passage, power generation, irrigation, and seasonal flow regulation [51]. Meanwhile, the dam has cut off the main stem, altering the spatiotemporal variation in material fluxes, with significant impacts on the riverine carbon and nitrogen cycles.

# 2. Sampling sites

The sampling sites shown in Fig. 1 included 43 permanent hydrological stations (blue open circles) where water quality was also conducted monthly. The streamwise length of the river from its furthest upstream station (Shigu) to its final downstream station (Xuliujing) near the estuary is about 4,300 km. Simultaneous sampling was arranged at corresponding stations (red solid circles in Fig. 1) in Spring and Autumn of 2014, respectively. In addition, monthly sampling of all elements was carried out from November 2014 to September 2015 at six major hydrological stations, namely, Panzhihua (PZH), Cuntan (CT), Yichang (YC), Wuhan (WH), Nanjing (NJ), and Xuliujing (XLJ) (purple solid circles in Fig. 1) along the Yangtze. Fig. 1 also shows the location of relevant sampling sites (yellow solid circles) where local monitoring has been undertaken [52, 53]. There were 24 sampling sites along the mainstream (M), including M1~M5 in Jinsha River in the plateau area; M6~M9 in the upper reach from Sichuan Basin to Chongqing; M10~M14 in the upper-middle reach comprising the plain and Dongting Lake, among which Miaohe station (M11) was 12 km upstream of the TGD whereas Huangling Temple station (M12) was located between the TGD and the Gezhou Dam; M15~M18 in the middle-lower reach between Dongting Lake and Poyang Lake and M19~M24 in the estuary reach of the lower Yangtze River.

# 3. Field sampling and analysis on dissolved and emitted greenhouse gases

To acquire matched information on hydrological parameters (including flow, velocity, runoff, temperature, etc.), water-quality (including biogenic elements, i.e., C, N and P), riverine dissolved and emitted CO_2_, CH_4_ and N_2_O at all 43 monitoring sites along the 4,300 km river continuum, two synchronous sampling campaigns were undertaken in March and October 2014 (Fig. 1). Furthermore, monthly sampling was carried out from October 2014 to September 2015 at six representative sites (PZH, CT, YC, WH, NJ, and XLJ) (Fig. 1).

Dissolved CO_2_, CH_4_, and N_2_O were analyzed using the headspace equilibration technique [54]. In brief, 75 ml of river water was collected in a 100 ml polypropylene (PP) syringe equipped with a two-way valve. Air bubbles were removed by tapping the syringe and expelling water while pointing the syringe upwards. Dissolved greenhouse gases (GHGs) were extracted by injecting 25 ml of ultra-high purity N_2_ into the syringe. The syringe was then shaken vigorously for 5 minutes to let CO_2_, CH_4_, and N_2_O diffuse out and equilibrate between the headspace and water phase. Finally, the headspace gas was transferred to a pre-vacuum glass storage vial equipped with a chlorobutyl septum. All samples were collected in triplicate.

CO_2_, CH_4_, and N_2_O emission rates were measured using the static floating chamber technique [54]. In short, several acrylic chambers, equipped with a vent hole and supported by a flotation collar, were tethered by a 3 m length of string to a boat that was allowed to drift freely during deployment. Two chambers were deployed to sample simultaneously at 10-minute intervals for 40 minutes. At the beginning of each deployment the chambers were positioned carefully on water surface and left for 15 s to equilibrate to atmospheric pressure, after which the vent hole was plugged with a rubber septum. Then 20 mL of gas was removed from the chamber using a 100 mL PP syringe equipped with a two-way valve. The gas samples were then transferred to pre-evacuated containers.

Concentrations of CO_2_, CH_4_, and N_2_O were determined using gas chromatograph; the detectors comprised a Thermal Conductivity Detector (TCD), Electron Capture Detector (ECD), and Flame Ionization Detector (FID), along with a HayeSep-Q, 5A molecular sieve and 5A molecular sieve columns. The oven temperature was set to 100 ^o^C, 50 ^o^C, and 280 ^o^C for CO_2_, CH_4_, and N_2_O respectively. System precision was less than 1% relative standard deviation at approximately ambient concentrations. Initial dissolved CO_2_, CH_4_, and N_2_O concentrations in water samples were determined using the method described by Beaulieu et al [52].

Bearing in mind the limited data available to establish the model (described below), we also utilized data accessible from previous studies conducted at certain sites along the Yangtze River to enhance the representativeness of our datasets [53, 54].

# 4. Historical data and *p*CO_2_ calculation

Routine monitoring campaign on water chemistry was conducted by the Changjiang Water Resources Commission on a monthly basis from 1990 to 2015. Information on pH, total alkalinity (TAlk), HCO_3_^-^, and water temperature (T) was acquired at 18 sampling sites along the mainstream (Supplementary Table 14). Thus, these datasets supported our estimation of historical *p*CO_2_ in the river through the CO2SYS program [55, 56] based on:

$$\text{CO}_{\text{2}}\text{+}\text{H}_{\text{2}}\text{O↔}\text{H}_{\text{2}}\text{CO}_{\text{3}}^{\text{*}}\text{↔}\text{H}^{\text{+}}\text{+}\text{HCO}_{\text{3}}^{\text{-}}\text{↔2}\text{H}^{\text{+}}\text{+}\text{CO}_{\text{3}}^{\text{2-}}$$

$${\text{ }\text{K}_{\text{0}}\text{=[H}}_{\text{2}}\text{CO}_{\text{3}}^{\text{*}}\text{]/[}\text{p}\text{CO}_{\text{2}}\text{]}$$

$$\text{K}_{\text{1}}\text{=}\text{[H}^{\text{+}}\text{]}\text{[HCO}_{\text{3}}^{\text{-}}\text{]/[}\text{H}_{\text{2}}\text{CO}_{\text{3}}^{\text{*}}\text{]}$$

$$\text{K}_{\text{2}}\text{=}\text{[H}^{\text{+}}\text{]}\text{[CO}_{\text{3}}^{\text{2-}}\text{]/[H}\text{CO}_{\text{3}}^{\text{-}}\text{]}$$

$${\text{ p}\text{CO}_{\text{2}}\text{=[H}}_{\text{2}}\text{CO}_{\text{3}}^{\text{*}}\text{]/}\text{K}_{\text{0}}\text{=}\text{[H}^{\text{+}}\text{]}\text{[HCO}_{\text{3}}^{\text{-}}\text{]/}\text{K}_{\text{0}}\text{K}_{\text{1}}$$

where $\text{H}_{\text{2}}\text{CO}_{\text{3}}^{\text{*}}$ is the sum of CO_2_aq and H_2_CO_3_. *K*_0_, *K*_1_ and *K*_2_ (p*K* = -log*K*) are thermodynamic reaction constants that are temperature (*T*) dependent and can be calculated from:

$$\text{p}\text{K}_{\text{0}}\text{=-7×}\text{10}^{\text{-5}}{\text{ }\text{T}}^{\text{2}}\text{+0.016}\text{T}\text{+1.11}$$

$$\text{p}\text{K}_{\text{1}}\text{=1.1×}\text{10}^{\text{-4}}{\text{ }\text{T}}^{\text{2}}\text{-0.012}\text{T}\text{+6.58}$$

$$\text{p}\text{K}_{\text{2}}\text{=9×}\text{10}^{\text{-5}}\text{ }\text{T}^{\text{2}}\text{-0.0137}\text{T}\text{+10.62}$$

This technique is well established for determining *p*CO_2_ in Chinese rivers [57, 58].

Most published *p*CO_2_ data were calculated from temperature, pH and total alkalinity (TAlk) [59, 60]. It was reported [61, 62] that the estimated *p*CO_2_ from PHREEQC program are very close to that derived from the CO2SYS with a minor difference <1~3 %. Overestimation of *p*CO_2_ occur only when lower buffering capacity of the carbonate system increases the sensitivity to TAlk in acidic and organic rich waters, or when organic acid anions significantly contribute to TAlk in water under low carbonate alkalinity and high DOC concentrations. For the Yangtze River with an annually averaged pH >7.85, such cases can hardly happen.

# 5. Artificial neural network model for estimation of dissolved CH_4_ and N_2_O

In river systems, CH_4_ is mainly produced through methanogenesis [63], which is primarily influenced by total organic carbon [64, 65] (TOC), water temperature [66-68] (T), dissolved oxygen [65] (DO), inorganic nitrogen [69-71] (DIN), Ph [72], and microbial populations and activity [63]. It is difficult to describe their relation by process-based models [73]. Similarly, N_2_O in rivers is also produced through sophisticated microbial processes, partly by nitrification (the aerobic oxidation of ammonium (NH_4_^+^) to nitrites (NO_2_^-^) and nitrates (NO_3_^-^)), and mainly by denitrification (the anaerobic dissimilatory reduction of NO_3_^-^ to N_2_) [74]. Primary factors include dissolved inorganic nitrogen (DIN, i.e., NO_3_^-^+ NH_4_^+^ + NO_2_^-^), dissolved oxygen (DO), water temperature (T), pH, water discharge, and microbial populations and activity [54, 75-78]. Given that NO_3_^-^, NH_4_^+^, and NO_2_^-^ are the substrates to produce N_2_O, they are often correlated with N_2_O concentration [54].

Here we use artificial neural networks (ANNs) to establish the nonlinear relationships among key variables to predict CH_4_ and N_2_O in waters [79]. The ANN model comprises of individual processing units named neurons that resemble neural activity. Each processing unit sums weighted inputs and then applies a function to the resultant to determine the output [80]. We use back-propagation networks (BPNs), which have hierarchical feed-forward network architecture. The classical BPN includes a single layer of input nodes, a layer of output processed elements, and at least one middle or hidden layer to capture nonlinearities in the input/output relationship. Using input and output sets with weightings to minimize errors between actual and predicted values, the BPNs are trained to learn the input/output relationship, and then validated against information from a separate dataset. Network training concludes when the mean squared error falls below a specified minimum, thus fixing the values for the weights [81]. At this point, the network is ready to provide fast response outputs to input data.

The Yangtze River passes through different geographical regions with varying anthropogenic activities. To reflect these differences along the long river, BPN models for dissolved CH_4_ and N_2_O are established respectively for three reaches, the upper, the middle, and the lower reaches of the Yangtze. According to statistical analysis, the input variables of the BPN model for dissolved CH_4_ (Supplementary Fig.17) included chemical oxygen demand (COD), water temperature (T), pH, dissolved oxygen, NH_4_^+^, and NO_3_^-^. Input variables of the BPN model for dissolved N_2_O estimation (Supplementary Fig.18) included NH_4_^+^, NO_2_^-^, NO_3_^-^, dissolved oxygen, water temperature, and pH. For wider applicability, unstructured data from other investigations were incorporated alongside our recently monitored data. By randomly choosing 80% of the collected data to train the BPN models, and the remaining 20% to test the models, reasonable estimates of dissolved CH_4_ and N_2_O were made in different reaches of the Yangtze River (details see Supplementary Table 14~15). Model validation (Supplementary Fig. 19 and Fig. 20) was performed in addition to sensitivity of the ANN model (Supplementary Fig. 15 and Fig. 16) by increase or decrease of individual variables at three levels (±10%, ±25%, and ±50%).

# 6. Estimation of CO_2_, CH_4_, and N_2_O emission rate and the uncertainty analysis

The CO_2_, CH_4_, and N_2_O emission rates could be determined from their dissolved concentrations by considering gas transfer velocities across the air-water interface. Multiplying the width of water surface at one representative station by the distance from this station to the next representative station, we can obtain the water-surface area of the river reach between the two hydrological stations. Multiplying this water-surface area by the emission rate at the upper station to yield the emission flux, and the total emission flux in the mainstream of the Yangtze River over 4300 km was derived by summing the emission fluxes at different river reaches.

Noting the requirement for synchronous data on hydrodynamics, four representative stations (Panzhihua (PZH), Cuntan (CT), Yichang (YC) and Wuhan (WH)) were selected to derive values for the gas transfer velocity. The mean value of the gas transfer velocities of PZH and CT represented the gas transfer velocity in upper reach of the Yangtze, and the corresponding mean value of YC and WH represented the transfer velocity in middle and lower reaches. Hence, CO_2_, CH_4_ and N_2_O emission rates were estimated at the corresponding stations along the Yangtze River. In this estimation, uncertainties might exist because the gas transfer velocity simply derived from some representative stations could be influenced by wind speed and flow rate. Performing 10,000 Monté Carlo permutations by setting a 10% increase/decrease in river area and gas transfer velocity, respectively, the resulting 10,000 estimates of GHGs flux varied 19% with the mean estimates, which should be applicable considering the complexity of the river system.

In the absence of full nitrogen records, a previous study estimated N_2_O emission based on a two-step approach [82] whereby riverine dissolved inorganic nitrogen (DIN) exports from the Yangtze River basin were estimated using the Global NEWS model, and then multiplied by the IPCC default emission flux (EF) value recommended as 0.0025 kg N_2_O-N/ kg N (within the range from 0.0005 to 0.025 kg N_2_O-N/ kg N) (*54*). Other studies have focused on the N_2_O emission at certain local areas over relatively short periods, such as values reported for particular sites along the Yangtze River [53, 78, 83]. However, any calculation based on the global default EF value would be likely to misestimate the actual N_2_O emission from diverse rivers [78, 84, 85]. For comparison, Supplementary Table 15 lists regression models for calculating dissolved N_2_O concentration from peer-reviewed publications.

The emission rate of GHGs across the air-water interface can be calculated using the following two-layer model for diffusive gas exchange [86]:

$$F=K_{w}\times\left( C_{obs}-C_{eq} \right) (1)$$

where *F* is the water surface emission rate of the GHG (μg m^-2^h^-1^), $C_{obs}$ is the concentration of the GHG in water column (μg L^-1^), and$C_{eq}$ is the equilibrium concentration of the riverine GHG with the atmosphere (μg L^-1^). In equation (1), *K_W_* is gas transfer velocity for the GHG (cm h^-1^), which can be determined by [86]

$$K_{W}=K_{600}\times{(\frac{S_{C}}{600})}^{-0.5} (2)$$

where *k*_600_ is the normalized *K_W_* value at 20℃ for freshwater, and S_C_ is the Schmidt number for GHGs, estimated using the following expressions from Wanninkhof [87].

$$S_{c}=1923.6-125.06T+4.3773T^{2}-0.085681T^{3}+0.00070284T^{4} (3-1)$$

$$S_{c}=1909.4-120.78T+4.1555T^{2}-0.080578T^{3}+0.00065777T^{4} (3-2)$$

$$S_{c}=2141.2-152.56T+5.8963T^{2}-0.12411T^{3}+0.0010655T^{4} (3-3)$$

in which *T* is water temperature (^o^C). Equations (3-1), (3-2) and (3-3) apply to CO_2_, CH_4_, and N_2_O, respectively. The normalized *K_W_* value at 20℃ for freshwater was estimated from

$$\text{k}_{\text{600}}\text{=1.0+1.719(}\frac{\text{v}}{\text{h}}\text{)}^{\text{0.5}}\text{+2.58}\text{μ}_{\text{10}} (4)$$

where ν is the river flow speed (m/s), h is water depth (m) and μ_10_ is wind speed 10 m above the water surface (m/s).

Herein, *k*_600_ is an important parameter for calculating gas emission rate from dissolved gas concentration [60, 88, 89]. There are two different scaling relationships between the turbulence-induced gas transfer velocity for low- and high-channel slope streams, indicating that gas exchange in streams exists in two states. Turbulent diffusion drives gas transfer velocity in low-energy streams, whereas turbulence entrains air bubbles in high-energy streams [90]. The gas transfer velocity is often quantified in streams, rivers, lakes, and oceans by injecting tracer gases, such as sulfur hexafluoride (SF_6_), propane or argon [91, 92]. In the present study, we use gas transfer velocity to calculate greenhouse gases fluxes across the air–water interface given that Yangtze River was low-energy stream (with the slope<4%) as reported in the previous study [93].

In general, *k*_600_ is mainly controlled by turbulence at air-water boundary layer [88, 94, 95]. Besides wind speed [86-88, 96, 97], other factors such as slope and velocity [88, 90, 94, 98, 99] may also influence the near-surface turbulence, and thus *k*_600_ is frequently parameterized as a function of wind speed, flow velocity, water depth, and bed roughness [90]. Based on comparison of the existing equations for *k*_600_ calculation (Supplementary Table 16), we used the formulas proposed for the Yangtze River [54, 78, 94].

Four representative stations (PZH, CT, YC and WH) were selected to derive the transfer velocity and to make uncertainty analysis [53, 78, 100-102]. Compared with the middle and lower reaches of the Yangtze River, the upper reach experienced varying landforms (Fig. 1). Water depth increases obviously from the upper to the lower reach (Supplementary Table 17). Thus, *k*_600_ in the upstream was represented by PZH and CT stations, and the averaged transfer velocities from PZH to CT were used for the upper reach [60]. For the middle and lower reaches from Yichang to Xuliujing, less difference was observed in flow velocity, water depth, and wind speed (Supplementary Table 17), and the average *k*_600_ from Yichang to Wuhan can reasonably represent the transfer velocity within the reaches.

# 7. Influence of tributary inputs on dissolved GHGs

In our field sampling campaign in 2014, we found dissolved GHGs concentrations were greater in the tributaries than those in the mainstream, like Jialing River in the upper reach and Huangbai River in the middle reach. However, tributary inputs had less impact on dissolved GHGs concentrations in the mainstream due to relatively smaller discharges. In the upper reach, the Jialing River entering into the Yangtze had higher *p*CO_2_ (874 μatm), dissolved CH_4_ (126 nmol/L), and dissolved N_2_O (0.45 μg/L) than those at the Cuntan station (599 μatm, 120 nmol/L, and 0.30 μg/L, respectively) located in the mainstream, but the smaller discharge from the tributary (1,905 m^3^/s) limited its influence on the GHGs concentrations in the mainstream. Similarly, the tributary Huangbai River in the middle reach had greater *p*CO_2_ (1753 μatm), dissolved CH_4_ (136 nmol/L), and dissolved N_2_O (0.92 μg/L) than those at Yichang station (1479 μatm, 78 nmol/L, and 0.39 μg/L respectively) in the mainstream, but the influx had little influence on GHGs concentrations in the mainstream due to smaller discharge (21 m^3^/s). In addition, the influx from the Poyang Lake had negligible influence on *p*CO_2_, dissolved CH_4_, and dissolved N_2_O at Jiujiang station in the lower reach of the mainstream.

# 8. CO_2_ decline in the middle reach

Compared with the significant decrease of *p*CO_2_ in the upper and lower reaches, *p*CO_2_ in the middle reach did not demonstrate apparent change (Figure. 3) due to “reservoir effects” combined with the spatial variations in geological and lithologic characteristics along the Yangtze. After impoundment of the TGR, higher trapping efficiency altered the carbon transportation regime of the river, leading to an increase in sediment deposition within the reservoir area. At global scale, the estimated organic carbon burial rate in reservoirs was 1,464 g CO_2_ m^–2^ yr^–1^, and the estimated annual organic carbon burial was about 0.06 Pg C (~40% of global inland water) [103-105]. The TGD resulted in a decline of sediment flux at Datong station from 210 Tg yr^-1^ in 2003 to 130 Tg yr^-1^ in 2008 (data obtained from Ministry of Water Resources of the People’s Republic of China, 2003–2010). The trapped organic carbon/sediments could provide respiration with substrate, leading to more CO_2_ production in the reservoir. Moreover, CO_2_ absorption in the surface due to photosynthesis and associated CO_2_ increased in the subsurface due to enhanced respiration may counteract each other (*78*), which partially explained why CO_2_ decrease in the middle reach was not so significant as expected after 2003.

Besides, the limestone was widely distributed in upper-middle reach, performed stronger buffer capacity than in lower reach with poor limestone [106-108]. Meanwhile, chemical erosion of carbonate minerals in the basin plays a major role in the DIC (mainly HCO_3_^-^) concentrations in river, decreasing from the upper reach to the river mouth [109, 110]. Average DIC over all cruises (field measurement and historical data, 1997–2008) was 1877 ± 354 μmol/L^60^ or 1900 μmol/L [110]. With increase in pH (Supplementary Fig. 13c), corresponding decrease in *p*CO_2_ occurred in the lower reach (Fig. 3a).

# 9. GHG emissions from reservoir, degassing and flooded area

Estimates of the GHGs emission associated with reservoirs would induce uncertainties to the total budget of the whole river system [111-113]. Besides the involved complexities of the biogeochemical processes, the absence of accurate models for assessing the GHGs footprint also contributes to this uncertainty [114]. Reservoirs have been considered carbon–neutral, carbon sinks or carbon sources (owing to increase GHG in the form of methane [115]). The surface areas and greenhouse gas fluxes in the mainstream of the river as well as in the reservoir itself before and after operation of TGD were shown in Supplementary Table 14 in addition to Supplementary Tables 19~20. Reservoirs could induce changes in flow regime, thermal stratification, water level, and land use [116, 117], companied with changes in the direction and magnitude of the associated C and N fluxes and thereby GHG emissions. Reservoirs increase water level and surface area, while the flooded soil respiration would enhance dissolved CO_2_ concentration [118-119]. Additional CO_2_ source may arise when significant amount of methane produced under anaerobic conditions is oxidized to CO_2_ by methanotrophs [120]. However, the increased CO_2_ could be offset by the lower mineralization rates under anoxic conditions in the case of reservoirs with anoxic hypolimnia [121]. If natural CO_2_ emissions are not discounted, only 25% could be resulted from the impoundment process, and current assessments of the CO_2_ footprint likely overestimate the net impact of reservoir CO_2_ emissions [114]. Previous study indicated that major tributaries entering the reservoir sometimes perform as a CO_2_ sink owing to TGR operation. These tributaries became more favorable to photosynthetic uptake of CO_2_ [122], contributed to the overall significant drop in annual averaged CO_2_ emission and fluxes to the ocean from the whole river after operation of TGD (Fig. 4b).

The flooded land due to reservoir would create new anoxic zone just below the soil-water interface. The complex biogeochemistry process leads to methane transformation and emission from aquatic systems via diffusion, ebullition, or degassing [123-125]. For hydropower reservoirs, the immediate GHGs degassing would occur due to abrupt changes in pressure and temperature as water passed through turbines and spillways [126]. Meanwhile, thermocline would show higher CH_4_ concentration due to possible reservoir hypolimnion, resulting in greater specific flux at the water-air interface and gaseous source. For example, methane concentration in the anoxic bottom layer (hypolimnion) was reported 150 times higher than that in the surface layer (epilimnion) [127]. Nevertheless, the water body in TGR was well mixed, given the relative higher water-flow velocity and the nearly constant turbulence in the reservoir, and minor contribution of GHG emissions was made from spillways and turbines and the uncertainty of GHG emissions from degassing could be neglected [128]. Moreover, bubbling CH_4_ emission, mostly occurred in dendritic reservoirs with vegetated littoral and deltaic deposition zones covered by shallow water (water depth < 10 m) [129], was highly restricted by the gorge topography of the valley-typed TGR. Another concern maybe CH_4_ emission from the ambient wetland due to increase of water level and surface area enhanced by TGD operation [130]. According to the measured CH_4_ emissions from fallow lands, croplands, and deforested lands in the TGR drawdown area and wetland ecosystems, CH_4_ emission rate in wetland area was comparably low [131-133]. Soft vegetation and organic matter accumulation would facilitate bubbling CH_4_ production, but conservation of water quality and clearance of dead plant matter and solid wastes prior to TGR impoundment limited the carbon substrate supplies for methanogens. Hence, the additional CH_4_ emission (0.25 Gg yr^-1^) due to the newly created marshes (100 km^2^) in the drawdown zone of TGR [131, 134] would not change our conclusions for the whole system budget (Supplementary Table 10 and 12, Figure 5).

In the perspective of N_2_O, the measured results suggested a decreased N_2_O emission rate immediately downstream the TGR [133]. The long-distance scouring downstream the dam modified habitats for heterotrophic denitrifies in riverbed, slowed down denitrification, and significantly restricted transformation from nitrate to N_2_O [135]. N_2_O degassing due to reservoir water undergoing rapid depressurization and/or strong aeration [113] was not quantified because of less contribution and absence of measured data.

# 10. Riverbed scouring in the downstream of the TGD

Long distance riverbed scouring was confirmed along the downstream of the TGD [136, 137], particularly in the middle reach (955km) of the Yangtze from Yichang to Hukou. Prior to impoundment of TGR, river channel in the middle reach maintained a general equilibrium between erosion and deposition, while an overall erosion of the bankfull channel (2002-2015) at the same reach was 16.478×10^8^ m^3^. For comparison, annual erosion and deposition of the bankfull channel from Yichang to Hukou prior to and post impoundment of the TGR was shown in Supplementary Fig. 21, together with the riverbed coarsening and the talweg deepening (Supplementary Fig. 22). Overall, the drastic sediment erosion and coarsening induced by TGD significantly altered the downstream habitats for microorganism to thrive and thereby microbial community and ecological functions [138-140].

# Supplementary Figures


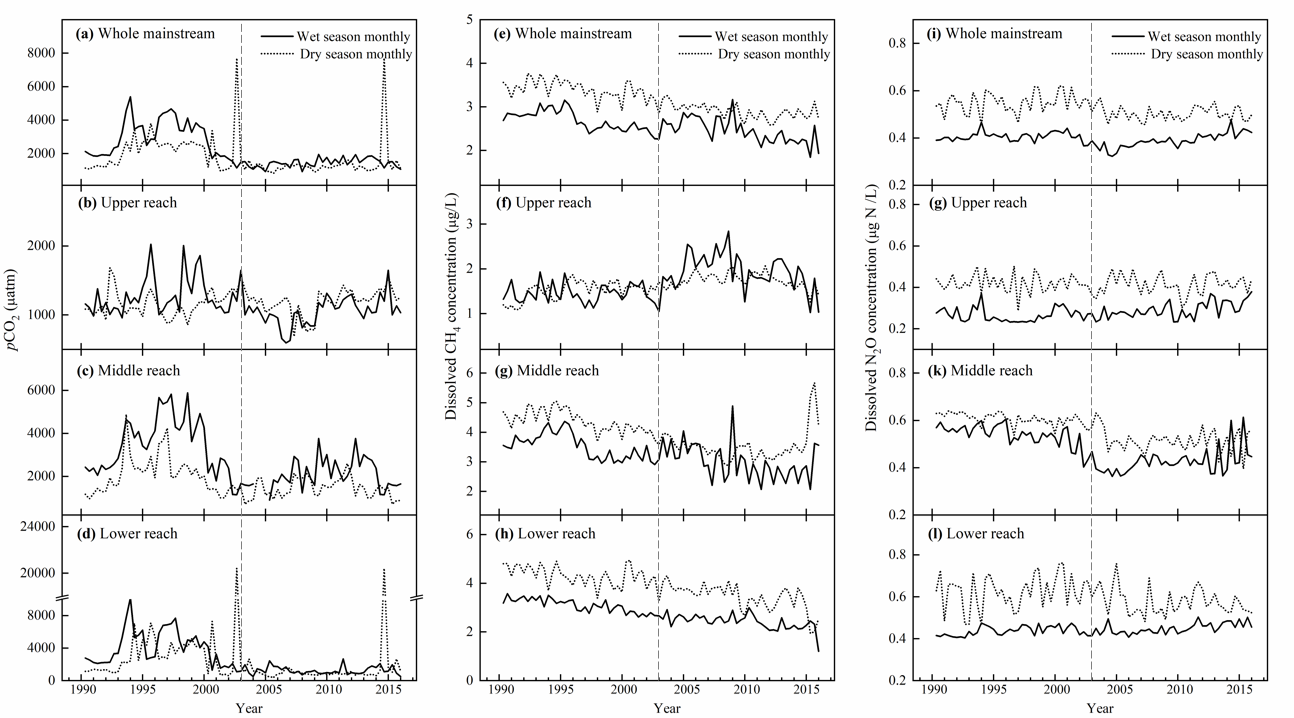


**Supplementary Fig. 1**. **Temporal variations in monthly averaged dissolved GHGs concentrations during wet and dry seasons over the 4,300 km continuum of the Yangtze River from 1990 to 2015**: monthly averaged *p*CO_2_ along the whole reach (**a**), the upper reach (**b**), the middle reach (**c**), and the lower reach (**d**); monthly averaged dissolved CH_4_ along the whole reach (**e**), the upper reach (**f**), the middle reach (**g**), and the lower reach (**h**); monthly averaged dissolved N_2_O along the whole reach (**i**), the upper reach (**j**), the middle reach (**k**), and the lower reach (**l**). Vertical dashed lines denote 2003, when TGD commenced operation.


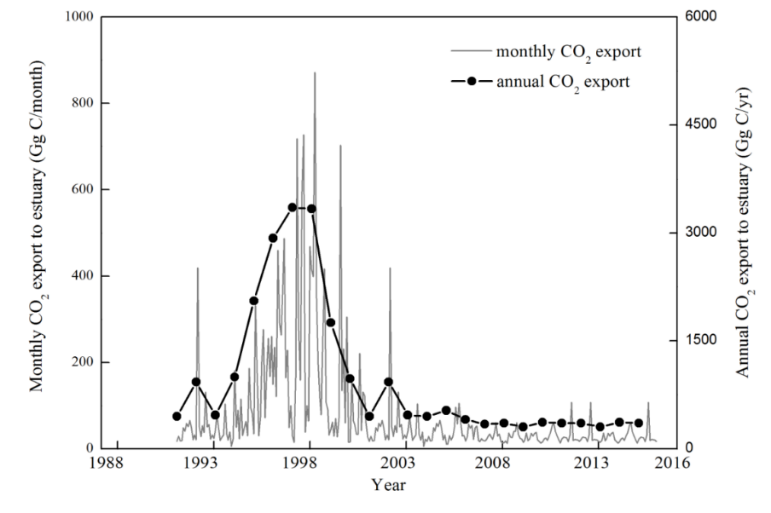


**Supplementary Fig. 2**. **Time series of monthly and annual fluvial export of CO_2_ from the Yangtze River to the East China Sea.**

**
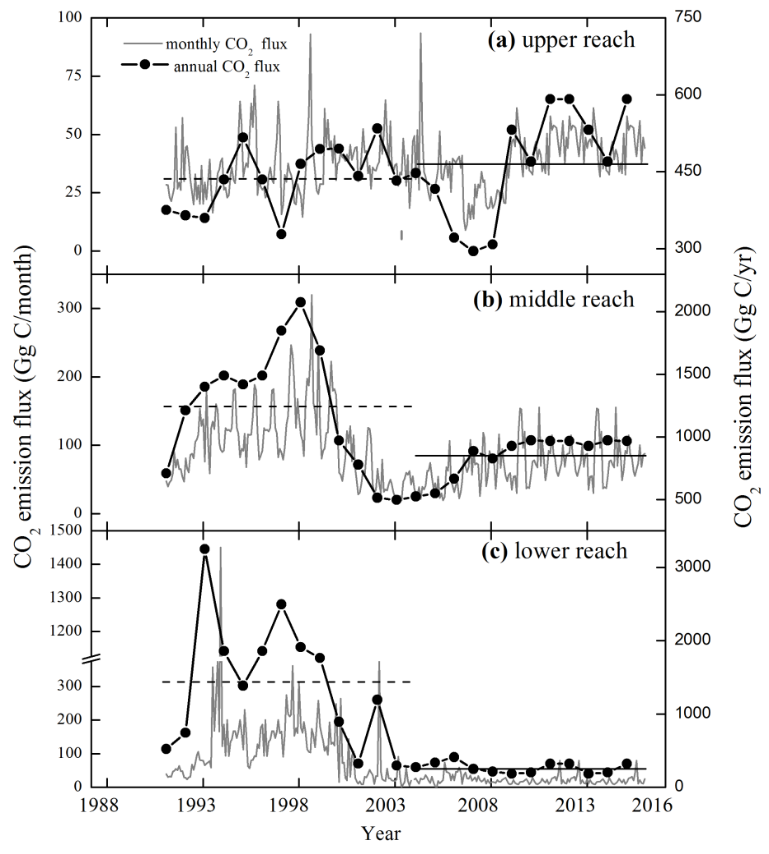
**

**Supplementary Fig. 3. Time series of monthly and annual** **CO_2_ emission fluxes from the Yangtze River mainstream.** CO_2_ emission fluxes from the upper reach (**a**), the middle reach (**b**), and the lower reach (**c**). The dashed and solid lines indicate mean annual values of CO_2_ emission flux before and after the impoundment of the TGD.

**
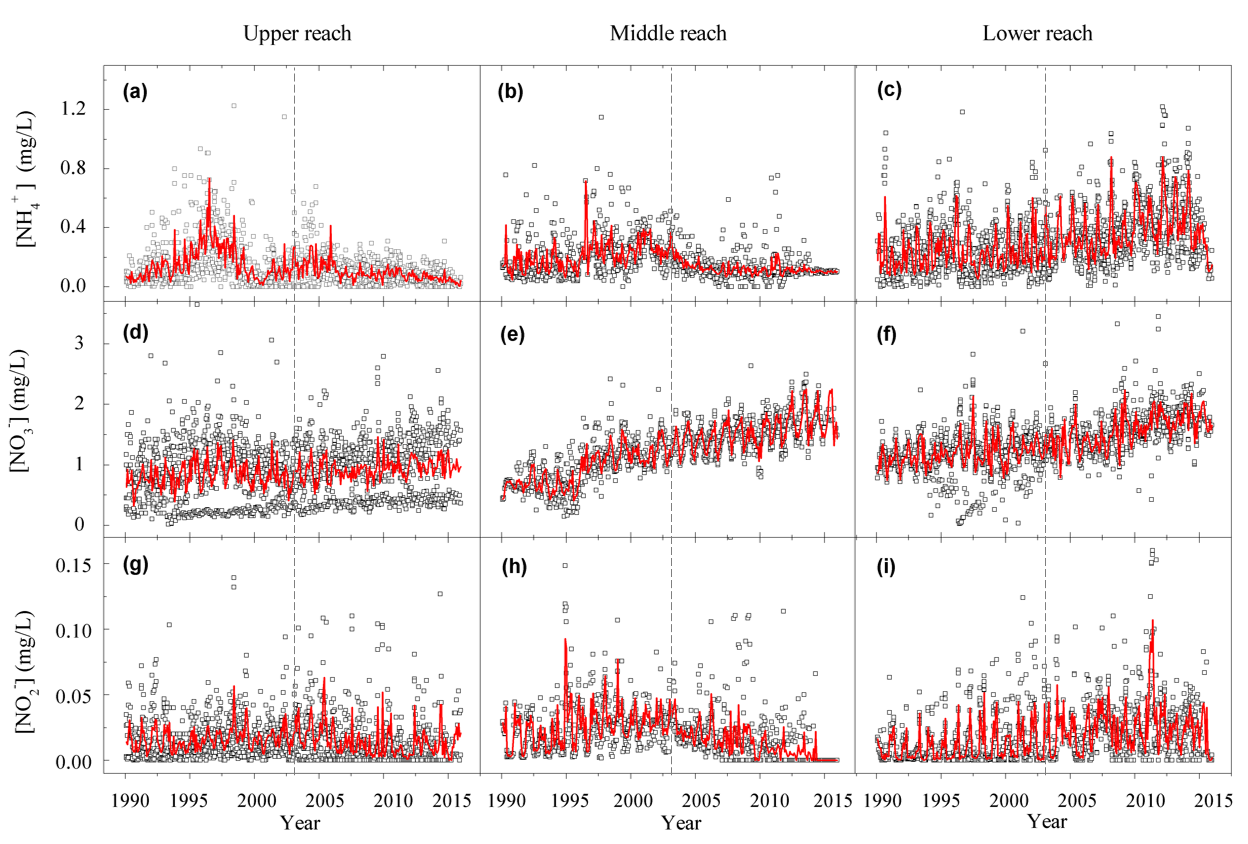
**

**Supplementary Fig. 4. Spatiotemporal variations in monthly NH_4_^+^, NO_3_^-^ and NO_2_^-^ concentrations in the upper, middle and lower reaches from 1990 to 2015 (total 312 months).** NH_4_^+^ in the upper reach (**a**), the middle reach (**b**) and the lower reach (**c**); NO_3_^-^ in the upper reach (**d**), the middle reach (**e**) and the lower reach (**f**); NO_2_^-^ in the upper reach (**g**), the middle reach (**h**) and the lower reach (**i**). Red lines show the monthly averaged concentration from all hydrological stations in 1990~2015, and open squares present the specific nitrogen concentration at hydrological stations. Vertical dashed lines denote 2003, when TGD commenced operation.


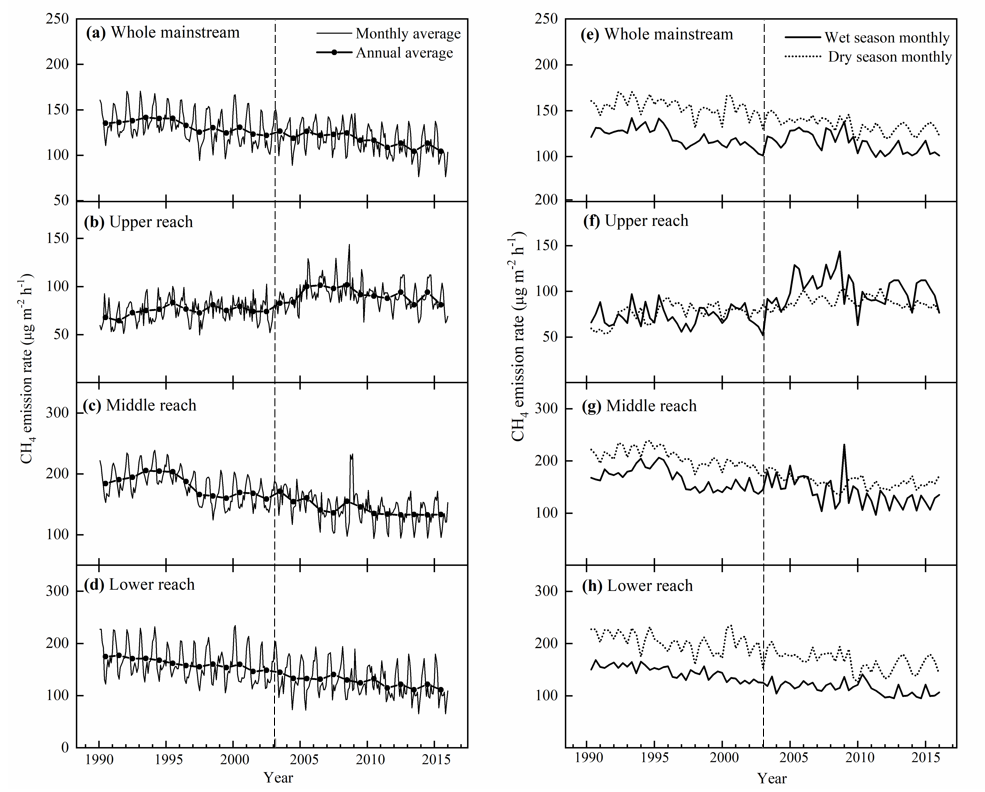


**Supplementary Fig. 5. Variations in monthly and annual averaged CH_4_ emission rates over the whole river continuum.** CH_4_ emission rate in the whole reach (**a**), the upper reach (**b**), the middle reach (**c**), and the lower reach (**d**) during the period of 1990~2015. Variations in averaged CH_4_ emission rate during wet and dry seasons in the whole river continuum (**e**), the upper reach (**f**), the middle reach (**g**) and the lower reach (**h**) from 1990 to 2015. The vertical dashed line indicates the year when TGD commenced operation.


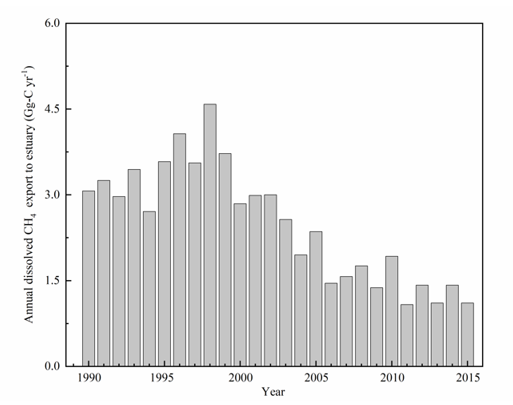


**Supplementary Fig. 6. Temporal variation in CH_4_ exported to the East China Sea.**


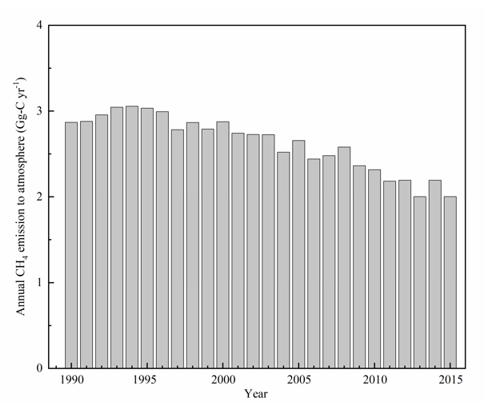


**Supplementary Fig. 7. Temporal variation in annual CH_4_ emission flux to atmosphere.**


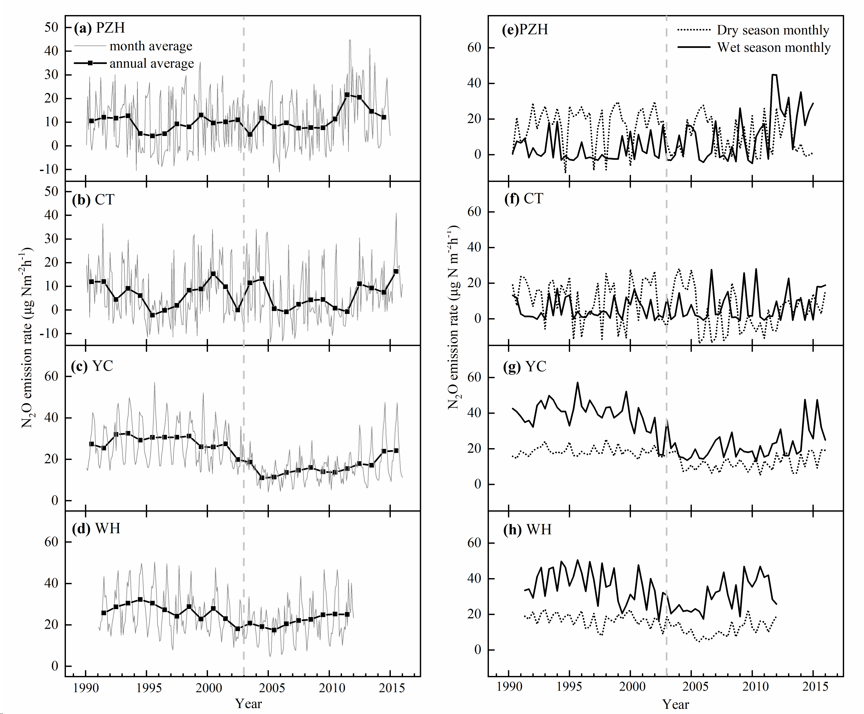


**Supplementary Fig. 8. Temporal variations in monthly and annual averaged N_2_O emission rates at four representative monitoring stations**: PZH (**a**), CT (**b**), YC (**c**), and WH (**d**) from 1990 to 2015; temporal variations in averaged N_2_O emission rates during wet and dry seasons at PZH (**e**), CT (**f**), YC (**g**), and WH (**h**) from 1990 to 2015. The vertical dashed line indicates the year when TGD commenced operation.


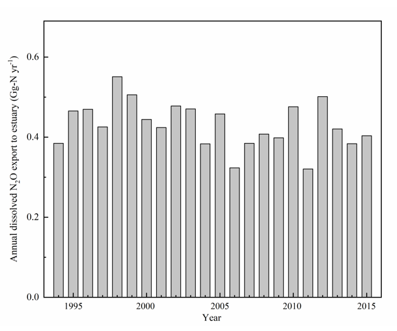


**Supplementary Fig. 9.** **Temporal variation in N_2_O exported to the East China Sea.**


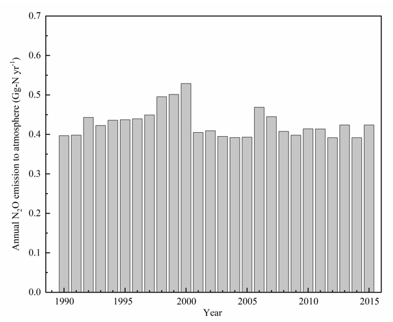


**Supplementary Fig. 10.** **Temporal variation in annual N_2_O emission flux to atmosphere.**

**
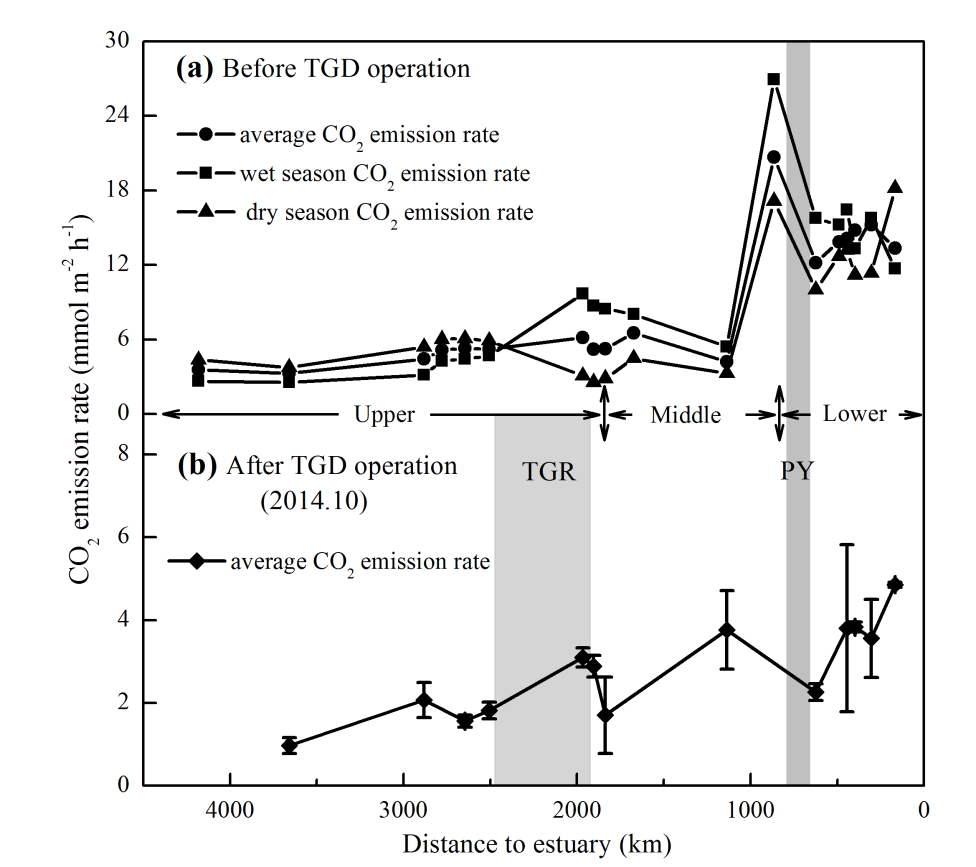
**

**Supplementary Fig. 11. Spatial variation in CO_2_ emission rate along the main stem before (a) and after (b) operation of the Three Gorges Dam.** Gray shaded regions represent the Three Gorges Reservoir and the inflow area of Poyang Lake.





**Supplementary Fig. 12**. **Temporal variation in monthly averaged (a) and seasonal averaged (b) dissolved N_2_O concentration at Yichang station.**


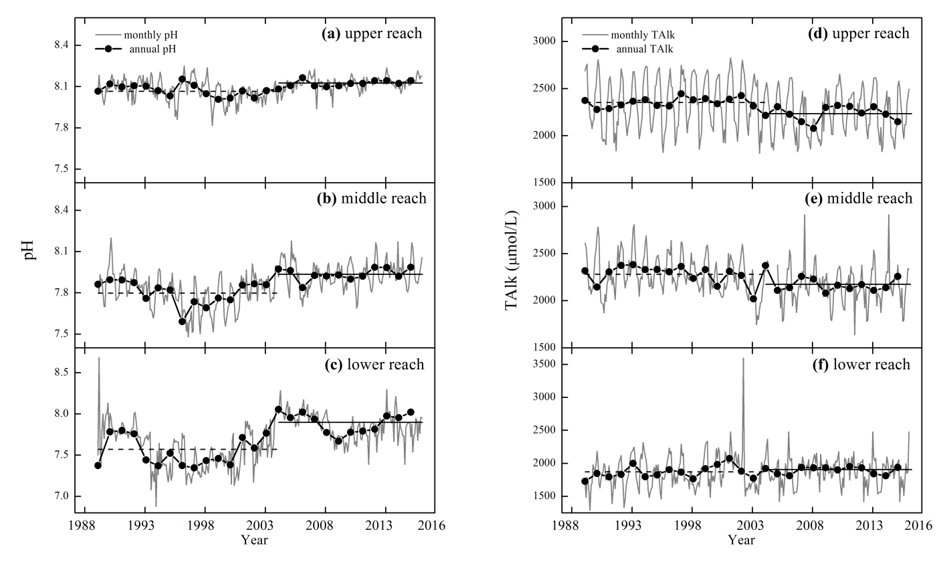


**Supplementary Fig. 13.** **Spatiotemporal variations in pH and TAlk in the Yangtze River mainstream.** (**a**) pH in the upper reach, (**b**) pH in the middle reach, and (**c**) pH in the lower reach. (**d**) TAlk in the upper reach, (**e**) TAlk in the middle reach, and (**f**) TAlk in the lower reach. The dashed and solid lines indicate the mean annual value of pH and TAlk before and after the operation of the TGD.

**
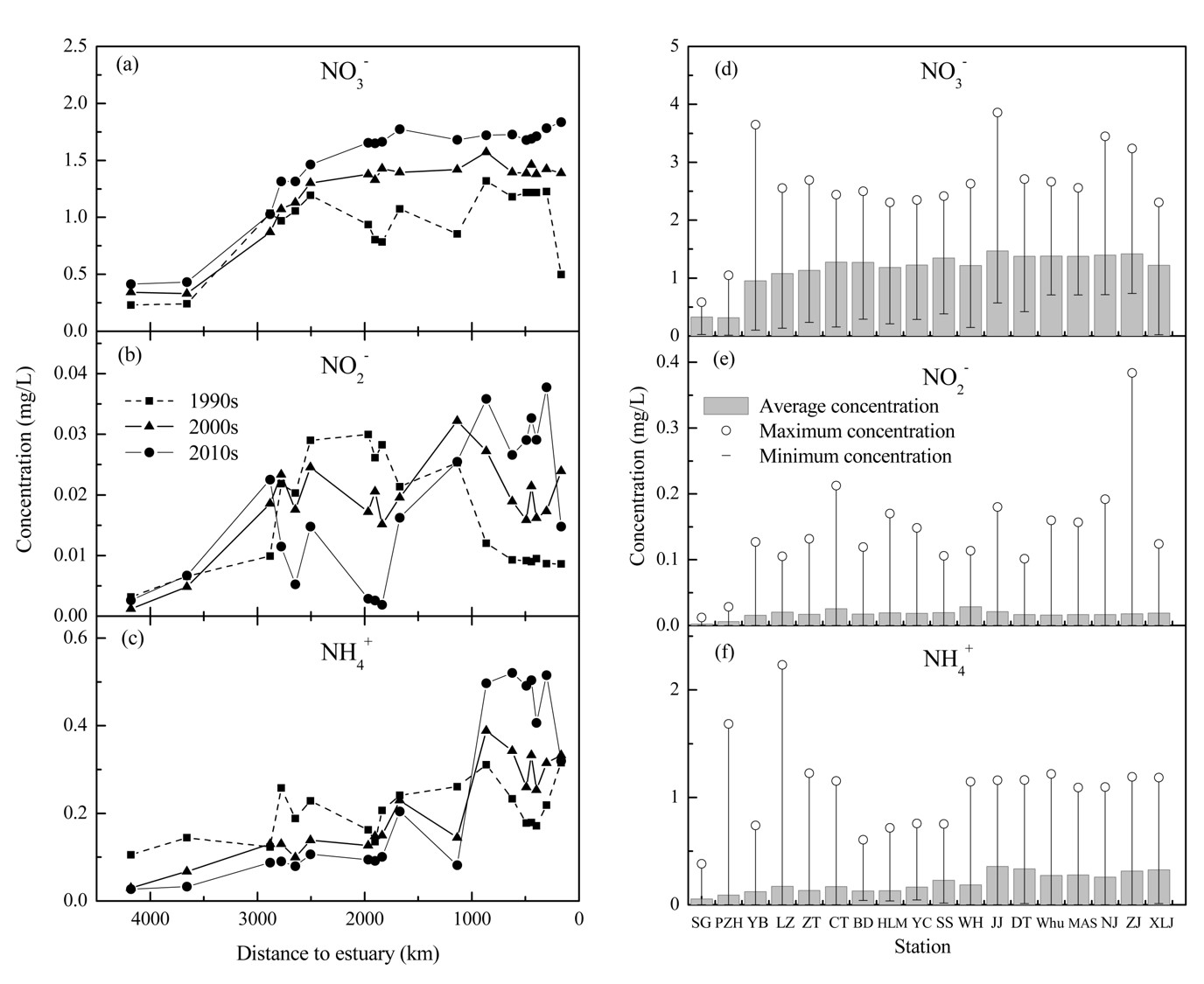
**

**Supplementary Fig. 14.** **Nitrogen levels along the mainstream of Yangtze River.** Averaged concentrations in NO_3_^-^ (**a**), NO_2_^-^ (**b**), and NH_4_^+^ (**c**) in 1990s, 2000s, and 2010s; monthly averaged, minimum, and maximum concentrations of NO_3_^-^ (**d**), NO_2_^-^ (**e**), and NH_4_^+^ (**f**) in the mainstream of Yangtze River during 1990~2015.





**Supplementary Fig. 15. Sensitivity of the dissolved CH_4_ model to changes in pH, water temperature (T), dissolved oxygen (DO), NH_4_^+^, NO_3_^-^, and chemical oxygen demand (COD).**





**Supplementary Fig. 16. Sensitivity of the dissolved N_2_O model to changes in pH, water temperature (T), dissolved oxygen (DO), NH_4_^+^, NO_3_^-^, and NO_2_^-^.**

**
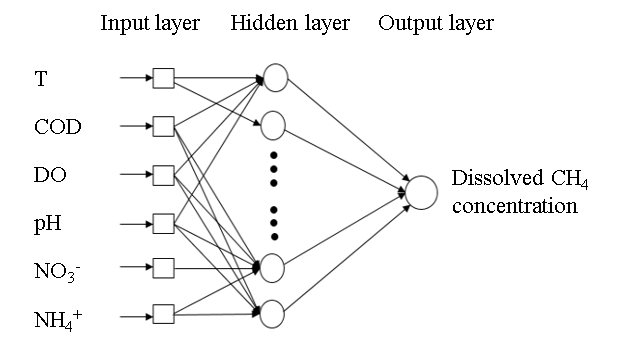
**

**Supplementary Fig. 17.** **Structure of the ANN model for dissolved** **CH_4_ concentration estimation.** It consists of one input layer of six vectors, one hidden layer and one output layer of dissolved CH_4_ concentration.


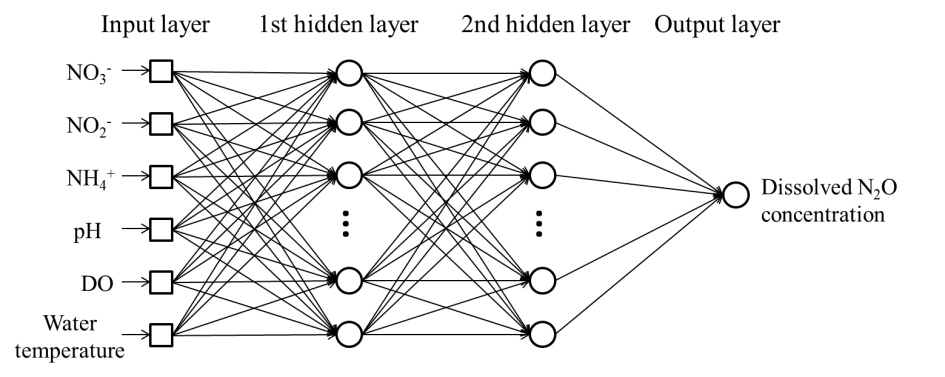


**Supplementary Fig. 18.** **Structure of the ANN model for dissolved N_2_O concentration estimation.** It consists of one input layer of six vectors, two hidden layers and one output layer of dissolved N_2_O concentration.





**Supplementary Fig. 19. Comparison of the modeled and observed concentration of CH_4_ concentrations for all data.** The black line is the 1:1 line.





**Supplementary Fig. 20. Comparison of the modeled and observed concentration of N_2_O concentrations for all data.** The black line is the 1:1 line.


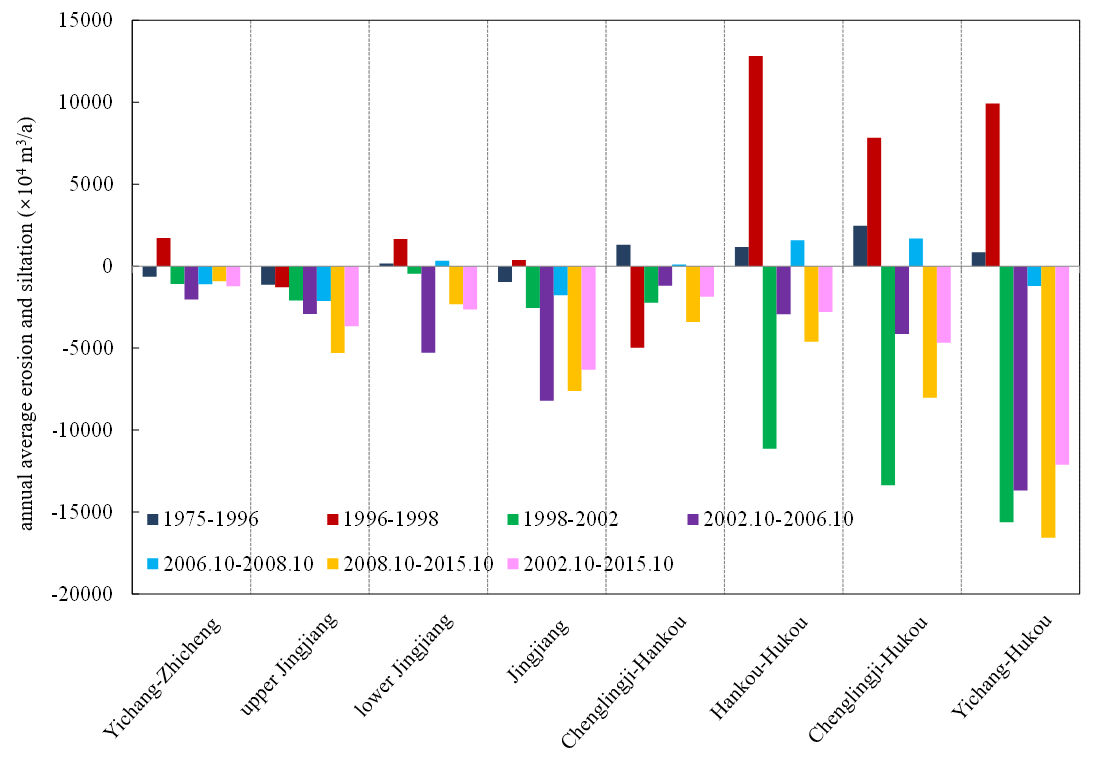


**Supplementary Fig. 21. The water-sediment annual erosion and siltation of the bankfull channel of the mainstream of Yangtze River from Yichang to Hukou before and after the impoundment of the Three Gorges Reservoir.**


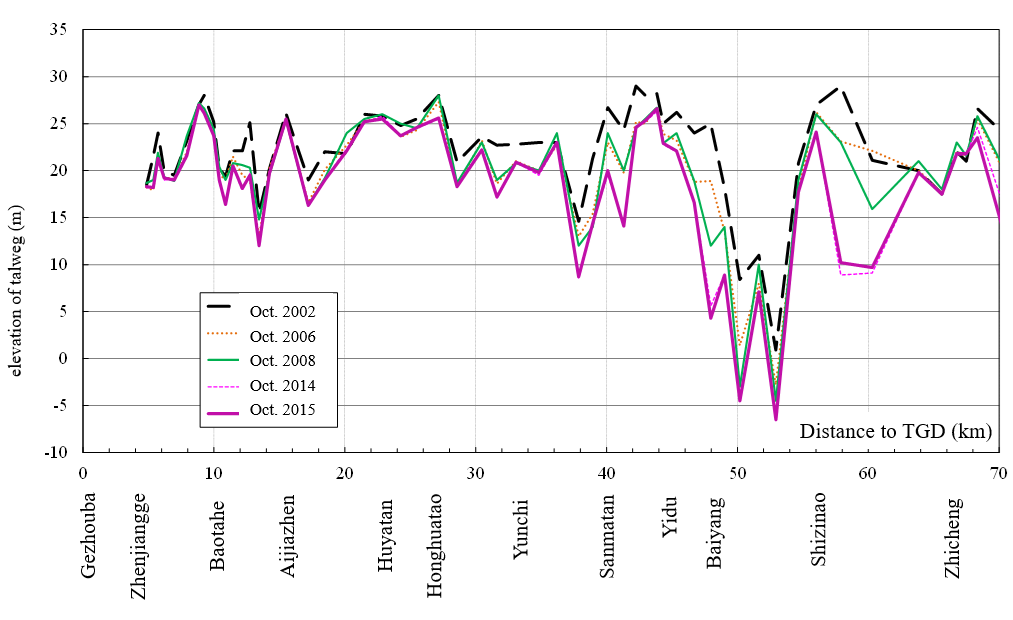


**Supplementary Fig. 22. The spatial variation of the talweg from Yichang to Zhicheng after the impoundment of the Three Gorges Reservoir**.

# Supplementary Tables

**Supplementary Table 1. Effects of dam scale on habitats and related materials**

| Dam name | Height  (m) | Period of interest | Influence distance  (km) | Material | Habitat |
| --- | --- | --- | --- | --- | --- |
| Glen Canyon Dam [141] | 220 | 1956-2000 | 141 | Sediment | Channel pattern |
| Three Gorges Dam [142] | 185 | 1981-2010 | 1,300 | Sediment | Channel erosion |
| Flaming Gorge Dam [143] | 153 | 1951-1996 | 105 | Sediment | Riverbed adjustments |
| Kurobe Dam [144] | 186 | 2007-2008 | 85 | Sediment,  Vegetation,  Nutrient (N, P) | Vegetative encroachment |
| Unazuki Dam [145] | 97 | 2007-2008 | 80 |  |  |
| Manwan Dam [146] | 132 | 1965-2003 | 401 | Suspended sediment | Reservoir capacity |
| Fortuna Dam [147] | 98 | 2003 | 40 | Greenhouse gas, organic matter | Thermodynamic stratification |
| Tucuruı´ Dam [148] | 78 | 1988-2005 | 300 | Sediment | Watercourse erosion |
| Livingston Dam [149] | 50 | 1924-1968,  1968-2014 | 120 | Sediment | Riverbed erosion,  channel geomorphology |
| Qingtongxia Dam [150] | 43 | 1976-2006 | 80 | Sediment | Land cover types |
| Samuel Dam [151] | 97 | 2004 | 40 | Greenhouse gas | Thermodynamic stratification |
| Petit Saut Dam [151] | 48 | 2003, 2005 | 40 | Greenhouse gas |  |
| Balbina Dam [151] | 33 | 2004 | 40 | Greenhouse gas |  |
| Hapcheon Dam [152] | 29 | 1983-2013 | 45 | Sediment, bed material | Bed degradation |
| Chijiawan Check Dam [153] | 13 | 2010-2012 | 5 | Sediment | Channel evolution |
| Elwha Dam [154] | 8 | 2010-2014 | 72 | Riverbed substrate | Bed material and habitat |

**Supplementary Table 2.** **Measured CH_4_ concentration at different stations of the Yangtze River (data from the literature)**

| No. | Site | Coordinate | Sampling information | | | | | | | |
| --- | --- | --- | --- | --- | --- | --- | --- | --- | --- | --- |
|  |  |  | Sample date | Months | CH_4_ | pH | T | DO | NH_4_^+^ | NO_3_^-^ |
| 1 | Yichang [52] | N30.70°; E111.28° | Jan, 2008;  Jun, 2010 | 2 | + | - | - | + | - | - |
| 2 | Shashi | N30.30°; E112.27° | Jan, 2008 | 1 | + | - | - | + | - | - |
| 3 | Wuhan | N30.62°; E114.32° | Jan, 2008 | 1 | + | - | - | + | - | - |
| 4 | Chenglingji | N29.27°; E113.09° | Jan, 2008 | 1 | + | - | - | + | - | - |
| 5 | Jiujiang | N29.75°; E116.00° | Jan, 2008 | 1 | + | - | - | + | - | - |
| 6 | Datong | N30.78°; E117.63° | Jan, 2008 | 1 | + | - | - | + | - | - |
| 7 | Wuhu | N31.45°; E118.33° | Jan, 2008 | 1 | + | - | - | + | - | - |
| 8 | Nanjing [52] | N32.17°; E118.95° | Jan, 2008 | 1 | + | - | - | + | - | - |
| 9 | Xuliujing [52] | N31.77°; E120.97° | Jul, 2009~Jun, 2010; | 12 | + | - | - | - | + | + |
|  |  |  | Jul, 2007~Sep, 2008 | 16 | + | - | + | - | - | - |

+ denotes data available for the corresponding index;

– denotes data not available for the corresponding index.

**Supplementary Table 3. Measured N_2_O concentration at different stations of the Yangtze River (data from literatures)**

| No. | Site | Coordinate | Sampling information | | | | | | | | |
| --- | --- | --- | --- | --- | --- | --- | --- | --- | --- | --- | --- |
|  |  |  | Sample date | Months | N_2_O | pH | T | DO | NH_4_^+^ | NO_3_^-^ | NO_2_^-^ |
| 1 | Nanxi [53] | N28.83°; E104.98° | 8/30~10/13, 2009 | 1 | + | - | - | - | - | - | - |
| 2 | Jiangjin | N29.30°; E106.28° | 8/30~10/13, 2009 | 1 | + | - | - | - | - | - | - |
| 3 | Shigu | N26.88°; E99.98° | 8/30~10/13, 2009 | 1 | + | - | - | - | - | - | - |
| 4 | Yichang | N30.70°; E111.28° | Jun, 2010 | 2 | + | - | - | - | - | - | - |
|  |  |  | Jan, 2008 | 1 | + | - | - | + | - | - | - |
| 5 | Zhicheng | N30.30°; E110.50° | Jan, 2008 | 1 | + | - | - | + | - | - | - |
| 6 | Shashi | N30.30°; E112.27° | Jan, 2008 | 1 | + | - | - | + | - | - | - |
| 7 | Jianli | N29.82°; E112.88° | Jan, 2008 | 1 | + | - | - | + | - | - | - |
| 8 | Wuhan | N30.62°; E114.32° | Jan, 2008 | 1 | + | - | - | + | - | - | - |
| 9 | Jiujiang | N29.75°; E116.00° | Jan, 2008 | 1 | + | - | - | + | - | - | - |
| 10 | Anqing | N30.50°; E117.10° | 8/30~10/13, 2009;  Jun, 2010 | 2 | + | - | - | - | - | - | - |
| 11 | Datong [53] | N30.78°; E117.63° | Jan, 2008; | 1 | + | - | - | + | - | - | - |
|  |  |  | Jun ~Dec, 2009 | 7 | + | - | - | - | - | + | - |
| 12 | Wuhu | N31.45°; E118.33° | Jan, 2008 | 1 | + | - | - | + | - | - | - |
| 13 | Nanjing | N32.17°; E118.95° | Jan, 2008 | 1 | + | - | - | + | - | - | - |
| 14 | Zhicheng | N32.18°; E119.67° | Jan, 2008 | 1 | + | - | - | + | - | - | - |
| 15 | Xuliujing  [53] | N31.77°; E120.97° | Jul, 2009~Jun, 2010; | 12 | + | - | - | - | + | + | + |
|  |  |  | Jan, 2008; | 1 | + | - | - | + | - | - | - |
|  |  |  | Jul, 2007~Sep, 2008; | 16 | + | - | + | - | - | - | - |
|  |  |  | Jan, 2010~Dec, 2011 | 24 | + | - | - | - | - | - | - |

+ denotes data available for the corresponding index; – denotes data not available for the corresponding index.

**Supplementary Table 4. Information on sampling sites along the Yangtze River**

| Mainstream | Sampling site | Distance to estuary | Coordinate | Years of historical data | Tributary | Sampling site | Coordinate | Years of historical data |
| --- | --- | --- | --- | --- | --- | --- | --- | --- |
| M1 | Shigu | 4,300 | E 99.58°; N 26.52° | 1982~2015, | T1 | Gaochang | E104.25°; N28.48° | 1980~2002, 2005~2014 |
| M2 | Panzhihua** | 3,706 | E101.42°; N26.34° | 1987~2014 | T2 | Wusheng | E106.16°; N30.21° | 1980~2012 |
| M3 | Xiluodu | 2,931 | E103.39°; N28.15° | 1987~2014 | T3 | Beibei | E106.27°; N29.48° | 1980~2014 |
| M4 | Pinshan | 2,811 | E104.10°; N28.38° | 2005~2011, 2014 | T4 | Xiaoheba | E105.50°; N30.11° | 1980~2014 |
| M5 | Xiangjiaba | 2,782 | E104.22°; N28.38° | 2005~2014 | T5 | Luoduxi | E106.35°; N30.21° | 1980~2014 |
| M6 | Yibin | 2,747 | E104.39°; N28.46° | 2005~2011, 2014 | T6 | Wulong | E107.45°; N29.19° | 1981~2014 |
| M7 | Luzhou | 2,612 | E105.33°; N28.53° | 1989~2014 | T7 | Xiaoxita | E111.18°; N30.46° | 1989~2004, 2006~2014 |
| M8 | Zhutuo | 2,521 | E105.51°; N29.01° | 1990~2014 | T8 | Yemingzhu | E111.17°; N30.44° | 1986, 1989~2014 |
| M9 | Cuntan** | 2,383 | E106.36°; N29.37° | 1986, 1988~2015 | T9 | Chenglingji | E113.8°; N29.25° |  |
| M10 | Badong | 1,852 | E110.24°; N31.02° | 1982~2014 | T10 | Nanzui | E112.17°; N29.4° |  |
| M11 | Miaohe | 1,795 | E110.54°; N30.53° |  | T11 | Zhouwenmiao | E112.3°; N28.54° |  |
| M12 | Huanglingmiao | 1,771 | E111.06°; N30.51° | 1989~2014 | T12 | Xiangyin | E112.52°; N28.40° |  |
| M13 | Yichang** | 1,737 | E111.16°; N30.41° | 1989~2014 | T13 | Baihe | E110.06°; N32.49° | 1986, 1992, 1994~2014 |
| M14 | Shashi | 1,577 | E112.15°; N30.17° | 1989~1995, 1997~2015 | T14 | Danjiangkou | E111.30°; N32.30° | 1986, 1992, 1994~2014 |
| M15 | Chenglingji | 1,333 | E113.09°; N29.27° | 1991~2002, 2004~2014 | T15 | Xiangyang | E112.08°; N32.01° | 1992, 1994~2014 |
| M16 | Luoshan | 1,303 | E113.19°; N29.39° | 1983~1987, 1991~1998 | T16 | Xiantao | E113.26°; N30.22° | 1991~1992, 1994~2014 |
| M17 | Wuhan** | 1,104 | E114.19°; N30.37° | 2000~2015 | T17 | Jijiazui | E114.13°; N30.34° | 1991~2000, 2002~2014 |
| M18 | Jiujiang | 863 | E115.59°; N29.44° | 2002~2014 | T18 | Taocha | E111.39°; N32.39° | 2004~2009, 2012~2014 |
| M19 | Datong | 632 | E117.38°; N30.46° | 1987, 1991~1992, | T19 | Hukou | E116.12°; N29.44° | 1994~2014 |
| M20 | Wuhu | 500 | E118.20°; N31.27° | 1994~2000, 2002~2014 |  |  |  |  |
| M21 | Maanshan | 456 | E118.28°; N31.46° | 1990~2003, 2005~2014 |  |  |  |  |
| M22 | Nanjing** | 382 | E118.56°; N32.10° | 1982~1984, 1989~2015 |  |  |  |  |
| M23 | Zhenjiang | 308 | E119.39°; N32.10° | 2005~2014 |  |  |  |  |
| M24 | Xuliujing** | 171 | E120.57°; N31.46° | 1990~2003, 2005~2015 |  |  |  |  |

** The stations where only monthly samplings made during October 2014 ~ September 2015.

Simultaneous samplings were made at all stations in March and October, 2014.

**Supplementary Table 5**. **Riverine carbon transport in several world’s largest rivers**

| River | Drainage area  (10^6^ km^2^) | discharge  (km^3^ yr^-1^) | DIC  (Tg C yr^-1^) | DOC  (Tg C yr^-1^) | POC  (Tg C yr^-1^) |
| --- | --- | --- | --- | --- | --- |
| Yangtze [155] | 1.80 | 900 | 20.2 | 1.17 | 1.88 |
| Mekong [58] | 0.80 | 470 | 4.46 | 2.20 |  |
| Yukong [156] | 0.86 | 205 | 4.96 | 1.58 | 0.75 |
| Mississippi [157] | 3.20 | 580 | 9.70 | 1.50 | 0.94 |
| Ottawa [158] | 0.15 | 61 | 0.52 |  |  |
| Amazon [159] | 6.11 | 6,590 | 34.80 | 26.40 | 13.20 |

**Supplementary Table 6. Riverine CO_2_ emission in selected river basins and regions worldwide**

| Region | Water surface area  (×10^3^ km^2^) | CO_2_ emission rate  (g C m^-2^ yr^-1^) | CO_2_ flux  (Pg C yr^-1^) |
| --- | --- | --- | --- |
| Globe [160] | 310~510 | 260~2,720 | 0.56 |
| Temperate zone [160] | 99~118 | 720~2,630 | 0.13 |
| Globe [161] | 536 | 3,358 | 1.80 |
| Globe [162] | 699~832 | 1,574 | 0.65 |
| Amazon basin [163] | 1,770 | 830 | 0.21 |
| Mississippi basin [157] | 9 | 1,182 | 0.01 |
| Yukong basin [156] | 10 | 750 | 0.008 |
| American rivers [163] | 40.60 | 882~4,008 | 0.10 |
| Temperate zone [163] | 230 | 2,370 | 0.50 |
| Xijiang River [57] | 0.19 | 830~1,560 | 2.22×10^-4^ |
| Ottawa River [158] | 0.92 | 1,700 | 1.56×10^-4^ |
| Mekong basin [58] | 7.95 | 852 | 68×10^-4^ |
| Yangtze River estuary [164] | 1.60 | 186~410 | (3.00~6.60)×10^-4^ |
| Yangtze River basin [165] | 90 | 170 | 0.02 |
| Yangtze River mainstream^a^ | 3.54 | 682 | 24.20×10^-4^ |
| Yangtze River basin^b^ | 90 | 287 | 0.03 |

a: CO_2_ emission rate was estimated based on pH and TAlk;

b: CO_2_ emission rate was measured by field sampling.

**Supplementary Table 7.** **Dissolved CH_4_ concentrations observed in some large rivers**

| River | Dissolved CH_4_  (mean) (ug/L) | | Sampling period | Time scale |
| --- | --- | --- | --- | --- |
| Yangtze River | | 1.57~2.76 (2.22) | Model estimation | Annual |
| Middle YR [53] | | 1.38~2.11 (1.65) | 2008.1 | Seasonal |
| Lower YR [53] | | 4.12~11.35 (7.74) | 2008.1 | Seasonal |
| Lower YR [53] | | 1.91~6.25 (4.08) | 2008.9 | Seasonal |
| Upper YR [53] | | 0.37~2.85 (1.44) | 2009.9~2009.10 | Seasonal |
| Middle-lower YR | | 1.79~4.78 (2.70) | 2003.4~2003.5 | Seasonal |
| Middle-lower YR [53] | | 0.72~3.08 (2.38) | 2009.9~2009.10 | Seasonal |
| Middle-lower YR [53] | | 0.23~3.18 (2.22) | 2010.6 | Seasonal |
| Pearl River [166] | | 1.01~31.92 (16.47) | 2003.9 | Seasonal |
| Alsea rivers [167] | | 0.35~11.66 (6.01) | 1979~1982 | Annual |
| Willamette [167] | | 2.48~4.77 (3.63) | 1979~1982 | Annual |
| Thames River [168] | | 4.40 | 1997.4 | Seasonal |
| Amazon River [169] | | 2.4~3.36 (2.88) | 1985~1987 | Annual |

**Supplementary Table 8.** **CO_2_, CH_4_ and N_2_O transfer rates and calculation parameters**

| Station | *u_10_* (m/s) | *h* (m) | *v* (m/s) | *T_w_*(℃) | *k*_CO2_  (cm/h) | *k*_CH4_  (cm/h) | *k*_N2O_  (cm/h) |
| --- | --- | --- | --- | --- | --- | --- | --- |
| Panzhihua  (PZH) | 1.3 | 5.6 | 2.0 | 16.1 | 4.9 | 4.8 | 13.8 |
| Cuntan  (CT) | 1.5 | 8.4 | 2.0 | 18.6 | 5.5 | 5.4 | 13.2 |
| Yichang  (YC) | 1.3 | 13.2 | 1.2 | 18.7 | 4.7 | 4.7 | 9.0 |
| Wuhan  (WH) | 1.4 | 11.8 | 1.1 | 18.5 | 4.9 | 4.9 | 9.5 |

**Supplementary Table 9.** **Dissolved N_2_O concentration observed in some large rivers**

| River | Dissolved N_2_O (mean) (μg/L) | Sampling period | Time scale |
| --- | --- | --- | --- |
| Yangtze River (YR) | 0.23-0.83 (0.45) | Model estimation | Annual |
| Yangtze River | 0.30-0.76 (0.47) | Oct, 2014 - Sep, 2015 | Seasonal |
| Middle-lower YR [78] | 0.64-0.81 (0.72) | Jun-Dec, 2009, 2011 | Diel/seasonal |
| Middle-lower YR [53] | 0.46-0.86 (0.62) | Jan, 2008 | Seasonal |
| Lower YR [54] | 0.34-0.72 | Jun-Dec, 2009 | Diel/seasonal |
| Lower YR [170] | 0.25-0.37 | Aug, Oct, 2002 | Seasonal |
| Yangtze River [53] | 0.19-0.34 (0.33) | Aug-Oct, 2009 | Seasonal |
| Amazon River [171] | 0.59 | Apr, 1982-Aug, 1985 | Seasonal |
| Adyar Rive [172] | 0.42-1.50 | Aug, 2003-Dec, 2004 | Seasonal |
| Hudson River [173] | 0.53 | May, 1998-Nov, 1999 | Seasonal |
| Millstone River [174] | 0.16-0.19 | Mar, May, 2002 | Diel |
| Iroquois River [174] | 0.19-0.39 | Apr, May, 2002 | Diel |
| Colne [175] | 1.24 | Aug, 2001- May, 2002 | Seasonal |
| Stour | 1.51 |  |  |
| Orwell | 1.68 |  |  |
| Deben | 1.34 |  |  |
| Trent | 1.21 |  |  |
| Ouse | 1.10 |  |  |
| Conwy | 0.67 |  |  |
| Mawddach | 0.62 |  |  |
| Dovey | 0.70 |  |  |

**Supplementary Table 10.** **Greenhouse gases fluxes before and after TGD operation (Gg /yr)**

| River reach | Before 2003 | | |  | After 2003 | | |  | Change (%) | | |
| --- | --- | --- | --- | --- | --- | --- | --- | --- | --- | --- | --- |
|  | CO_2_ | CH_4_ | N_2_O |  | CO_2_ | CH_4_ | N_2_O |  | CO_2_ | CH_4_ | N_2_O |
| Upper reach | 409.17 | 0.38 | 0.05 |  | 400.91 | 0.49 | 0.04 |  | -2.02 | +29.26 | -26.90 |
| Middle reach | 553.46 | 1.27 | 0.20 |  | 557.54 | 1.00 | 0.18 |  | +0.74 | -21.33 | -8.51 |
| Lower reach | 2243.07 | 1.60 | 0.19 |  | 498.30 | 1.22 | 0.19 |  | -77.78 | -24.26 | -0.81 |
| Whole river (emission) | 3205.70 | 3.25 | 0.44 |  | 1456.75 | 2.71 | 0.41 |  | -54.56 | -16.85 | -7.01 |
| Whole river (to estuary) | 1863.24 | 3.09 | 0.46 |  | 391.80 | 1.55 | 0.41 |  | -78.97 | -49.84 | -9.45 |

- “-” denotes a decrease of GHG flux after operation of TGD since 2003.

**Supplementary Table 11. Concentrations of Chl *a* in tributaries of the Three Gorges Reservoir**

| River | Drainage area (km^2^) | Length (km） | Monitoring period | Concentration of  Chl *a* (μg/L) |
| --- | --- | --- | --- | --- |
| Meixi River [176] | 1,932 | 117 | 2013 | 0.38-28.13 |
|  |  |  | March, 2013 | <2.00 |
|  |  |  | April-July, 2013 | 5.00 |
|  |  |  | September, 2013 | 25.00 |
|  |  |  | December, 2013 | <1.00 |
| Xiangxi River [177, 178] | 3,099 | 94 | June, 2010 | 16.38-30.28 |
|  |  |  | January, 2010 | 21.13, with the maximum of 227.84 |
|  |  |  | February, 2010 | 22.52 |
|  |  |  | June, 2008 | algal bloom area, 65.84 |
|  |  |  |  | none-algal bloom area, 20.85 |
|  |  |  | July, 2008 | algal bloom area, 67.93 |
|  |  |  |  | none-algal bloom area, 18.12 |
| Tangxi River [179] | 1,707 | 104 | May, 2006 | backwater area, 20.00 |
| Xiaojiang River [180] | 5,225 | 183 | May, 2006 | backwater area, 28.90 |
|  |  |  | August, 2010 | 30.00 |
|  |  |  | October, 2010 | 50.00 |
|  |  |  | March, 2011 | 285.00 |
|  |  |  | May, 2011 | 100.00 |
| Modaoxi River [179] | 3,092 | 191 | May, 2006 | backwater area, 26.00 |

**Supplementary Table 12.** **Increased GHGs emission derived from the reservoir itself** **under** **practical scenarios for TGD operation and its offset through reduction of emission fluxes downstream Yangtze River.**

| Practical scenarios for TGD operation | TGR area (km^2^) | Enlargement GHGs emission caused by TGR (Gg/yr) | Offset distance downstream the TGD (km) | Net reduction in GHGs emission in the entire Yangtze (%) |
| --- | --- | --- | --- | --- |
| High water level  (175 m) | 1084 | CO_2_: 3.4×10^2^ | CO_2_: 819 | CO_2_: 38.43 |
|  |  | CH_4_: 3.7×10^-1^ | CH_4_: 180 | CH_4_: 14.51 |
|  |  | N_2_O: 1.0×10^-2^ | N_2_O: 53 | N_2_O: 0.21 |
| Annual average water level (163 m) | 944 | CO_2_: 2.7×10^2^ | CO_2_: 801 | CO_2_: 40.90 |
|  |  | CH_4_: 2.9×10^-1^ | CH_4_: 153 | CH_4_: 16.68 |
|  |  | N_2_O: 6.3×10^-3^ | N_2_O: 36 | N_2_O: 1.16 |
| Low water level  (145 m) | 734 | CO_2_: 1.8×10^2^ | CO_2_: 766 | CO_2_: 44.36 |
|  |  | CH_4_: 1.8×10^-1^ | CH_4_: 124 | CH_4_: 19.70 |
|  |  | N_2_O: 7.2×10^-4^ | N_2_O: 18 | N_2_O: 2.50 |

**Supplementary Table 13. Newly created pathways of GHG emissions from the TGR after impoundment**

|  | CO_2_ (Gg/yr) | CH_4_ (Mg/yr) | N_2_O (Mg/yr) |
| --- | --- | --- | --- |
| Reservoir drawdown area | 82.6 (70.5~110.2) | 53.9 (44.3~64.9) | 1.85 (1.38~2.34) |
| Ebullition | 1.08×10^-3^ (8.30×10^-4^ ~0.97) | 5.65 (0.83~12.5) | 0.11 (0.03~1.27) |
| Degassing | 6.3 (4.5~7.9) | 14.0 (11.8~16.1) | 0.41 (0.25～1.38) |

**Supplementary Table 14. Details of BPN models used to estimate dissolved** **CH_4_ concentrations in different reaches of the Yangtze River**

| River reach | Neutrons of the hidden layer. | MSE |
| --- | --- | --- |
| Upper | 10 | 0.028 |
| Middle | 20 | 0.048 |
| Lower | 13 | 0.019 |

*MSE refers to the minimum mean square error obtained through repeating and changing the training and testing processes.

**Supplementary Table 15. Details of BPN models used to estimate dissolved N_2_O concentrations in different reaches of the Yangtze River**

| River reach | Neutrons of the  1st hidden layer | Neutrons of the  2nd hidden layer | MSE |
| --- | --- | --- | --- |
| Upper | 6 | 6 | 0.00096 |
| Middle | 11 | 14 | 0.00187 |
| Lower | 14 | 2 | 0.00199 |

*MSE refers to the minimum mean squared error obtained through repeating and changing the training and testing processes.

**Supplementary Table 16.** **Historical data on water chemistry at stations along the Yangtze**

| No. | Mainstream | pH | TAlk  (μmol/L) | *p*CO_2_  (μatm) | Historical data |
| --- | --- | --- | --- | --- | --- |
| 1 | Shigu | 8.00 ~ 8.54 | 1,680 ~ 3,180 | 341 ~ 1,385 | 1989.01 ~ 1993.12  1995.01 ~ 1999.12  2000.01 ~ 2005.12 |
| 2 | Panzhihua | 8.00 ~ 8.60 | 1,680 ~ 3,328 | 352 ~ 1,640 | 1989.01 ~ 2005.12 |
| 6 | Yibin | 7.80 ~ 8.40 | 1,610 ~ 3,956 | 485 ~ 3,102 | 1989.01 ~ 2005.12 |
| 7 | Luzhou | 7.60 ~ 8.30 | 1,705 ~ 2,951 | 595 ~ 3,519 | 1990.05 ~ 2005.12 |
| 8 | Zhutuo | 7.57 ~ 8.37 | 1,659 ~ 3,083 | 588 ~ 3,463 | 1989.01 ~ 2012.12 |
| 9 | Cuntan | 7.57 ~ 8.43 | 1,435 ~ 2,901 | 430 ~ 3,077 | 1989.01 ~ 1995.12  1997.01 ~ 2012.12 |
| 10 | Badong | 7.40 ~ 8.20 | 1,854 ~ 2,914 | 893 ~ 5,343 | 1997.04 ~ 1999.12 |
| 12 | Huanglingmiao | 7.40 ~ 8.20 | 1,870 ~ 3,183 | 886 ~ 5,322 | 1989.04 ~ 1999.12 |
| 13 | Yichang | 7.40 ~ 8.23 | 1,830 ~ 3,261 | 894 ~ 5,274 | 1989.01 ~ 1995.12  1997.01 ~ 1999.12 |
| 14 | Shashi | 7.23 ~ 8.32 | 1,918 ~ 2,819 | 669 ~ 9,079 | 1992.01 ~ 2002.12 |
| 17 | Wuhan | 7.50 ~ 8.20 | 1,638 ~ 2,910 | 713 ~ 3,950 | 1991.01 ~ 1992.12  1994.01 ~ 1995.12  1997.01 ~ 2000.12  2002.01 ~ 2003.12  2005.01 ~ 2011.12 |
| 18 | Jiujiang | 7.07 ~ 7.90 | 1,263 ~ 2,833 | 1,335 ~ 13,265 | 1990.04 ~ 1999.12 |
| 19 | Datong | 6.75 ~ 8.68 | 1,277 ~ 2,405 | 158 ~ 22,423 | 1989.01 ~ 1999.12 |
| 20 | Wuhu | 7.00 ~ 8.08 | 1,416 ~ 2,647 | 784 ~ 12,102 | 1989.04 ~ 1999.12 |
| 21 | Maanshan | 7.00 ~ 8.00 | 1,295 ~ 2,398 | 865 ~ 11,620 | 1989.04 ~ 1999.12 |
| 22 | Nanjing | 6.33 ~ 8.10 | 1,231 ~ 2,517 | 855 ~ 59,823 | 1989.01 ~ 1999.12 |
| 23 | Zhenjiang | 6.53 ~ 8.00 | 1,242 ~ 2,398 | 1,014 ~ 38,744 | 1989.04 ~ 1999.12 |
| 24 | Xuliujing | 6.75 ~ 8.56 | 1,345 ~ 3,596 | 308 ~ 20,421 | 1994.01 ~ 2011.12 |

**Supplementary Table 17.** **Existing regression models used for calculation of dissolved N_2_O concentration**

| No. | Model | R^2^ | P | Study area | Time |
| --- | --- | --- | --- | --- | --- |
| 1 [78] | $\Delta N_{2}O=-0.12[DO]+0.15[NO_{3}^{-}]+7.2$(runoff rivers) | 0.44 | <0.001 | YR: Datong; Maanshan, Anqing, Hankou, Wanzhou, Cuntan; HR: Xiantao; JLR: Beibei | Jun-Dec, 2009 and Oct, Nov, 2011 |
|  | $\Delta N_{2}O=0.55([DOsat]-[DOmeas])-1.37$(runoff rivers) | 0.17 | <0.001 |  |  |
|  | $\Delta N_{2}O=0.14[DIN]-0.05[DO]+0.52$(runoff rivers + urban rivers) | 0.30 | 0.002 |  |  |
| 2 [54] | $\log[N_{2}O]=0.69\times\log[NO_{3}^{-}]-0.39$ | 0.52 | <0.0001 | YR: Anqing, Datong, Maanshan | Jun-Dec, 2009 |
|  | $[N_{2}O]=0.06[DO]-0.12$ | 0.39 | <0.0001 |  |  |
| 3 [53] | $[N_{2}O]=0.074[DIN]+0.23716$ | 0.62 | <0.0001 | YR: mainstream and tributaries | Aug-Oct, 2009 |
| 4 [181] | $[N_{2}O]=(0.651[NO_{3}^{-}]+1.39)\times[N_{2}Oequ]$ | 0.335 | 0.000 | YR: Datong; Maanshan, Anqing, Hankou, Wanzhou, Cuntan; HR: Xiantao; JLR: Beibei | Jun-Dec, 2009;  Nov-Dec, 2011 |
|  | $[N_{2}O]=(-1.84[NH_{4}^{+}]+2.74)\times[N_{2}Oequ]$ | 0.159 | 0.001 |  |  |

*[*DO*] is the dissolved oxygen concentration (mg L^-1^), [*DOsat*] is the DO saturation concentration (mg L^-1^), [*DOmeas*] is the measured DO concentration (mg L^-1^);

[*NO_3_^-^*] is the dissolved NO_3_^-^ concentration (mg N L^-1^), [*NH_4_^+^*] is the dissolved NH_4_^+^ concentration (mg N L^-1^), [*DIN*] is the concentration of dissolved inorganic nitrogen (DIN) (mg N L^-1^);

[*N_2_O*] is the dissolved N_2_O concentration (μg N L^-1^), [*N_2_Oequ*] is the equilibrium N_2_O concentration of river water with the atmosphere (μg N L^-1^), ∆*N_2_O* is air-water N_2_O concentration gradient (μg N L^-1^) defined by $\Delta N_{2}O=[N_{2}Omeas]-[N_{2}Oeqa]$ where $[N_{2}Omeas]$ is the measured N_2_O concentration (μg N L^-1^);

YR, HR, and JLJ refer to the Yangtze River, Hanjiang River, and Jialingjiang River, respectively.

**Supplementary Table 18. Existing empirical formulas for calculating *k*_600_**

| Formula | Notation | Reference |
| --- | --- | --- |
| $k_{600}=0.31u_{10}^{2}$ $k_{600}=0.39u_{10}^{2}$ $k_{600}=0.251u_{10}^{2}$     | $v$: flow velocity  $h$: water depth  $u_{10}$: wind speed at 10 m above water surface | Liss & Merlivat, 1986 [96]; Garnier et al., 2009 [182]; Zhang et al., 2010 [83]; Clough et al., 2011 [183]; Wanninkhof, 2014 [87]; Chen et al., 2015 [77] |
| $k_{600}=1.0+1.719(v/h)^{0.5}+2.58u_{10}$ |  | Borges et al., 2004 [94]; Yan et al., 2012 [54]; Garnier et al., 2009 [182] |
| $k_{600}=1.91e^{0.35u_{10}}$ |  | Raymond & Cole, 2001 [85]; Zhang et al., 2010 [101] |
| $k_{N_{2}O}=0.913k_{O_{2}}$ | $k_{O_{2}}$: gas transfer velocity for O_2_ | Holmén & Liss, 1984 [184]; Reay et al., 2003 [185]; Clough et al., 2006 [84] |
| PoRGy model | Computer program model | Venkiteswaran et al., 2007 [186]; Rosamond et al., 2011 [187]; Baulch et al., 2012 [188]; Venkiteswaran et al., 2014 [189] |

**Supplementary Table 19. *k*_600_ equations used at monitoring sites along the Yangtze river in the present study and published literatures**

| Station | Reach | Monitoring period | V (m/s) | H (m) | u_10_ (m/s) | *k*_600_  (cm/h) | *k*_600_-mean  (cm/h) | Reference |
| --- | --- | --- | --- | --- | --- | --- | --- | --- |
| Shigu | Upper | 2006~2015 | 0.56~2.33 | 2.24~7.82 | 1.50~7.30 | 5.72~20.68 | 9.07 | This study |
| Panzhihua** | Upper | 2006~2015 | 1.23~3.39 | 1.95~11.77 | 0.70~3.08 | 3.79~8.24 | 5.93 | This study |
| Beibei | Upper | 2011.10/12 | 0.17~0.2 | 26 | 0.7~0.9 | 3.10~3.52 |  | Wang et al., 2015 [78] |
| Cuntan** | Upper | 2006~2015 | 0.25-2.29 | 3.90~18.69 | 0.30~1.94 | 2.04~12.40 | 6.12 | This study |
| Wanzhou | Upper | 2011.10/12 | 0.41~0.53 | 90 | 0.6~0.7 | 2.91~3.10 |  | Wang et al., 2015 [78] |
| Yichang** | middle | 2006~2015 | 0.61~2.42 | 9.25~20.71 | 0.50~2.17 | 2.24~12.58 | 5.93 | This study |
| Xiantao | middle | 2011.10/12 | 1~1.12 | 5.3 | 0.9~1.1 | 4.55~4.60 |  | Wang et al., 2015 [78] |
| Hankou | middle | 2011.10/12 | 1.6~1.8 | 24 | 0.7~0.8 | 3.10~3.95 |  |  |
| Wuhan** | middle | 2006-2015 | 0.81~1.69 | 6.21~19.42 | 0.30~4.30 | 2.35~12.61 | 5.96 | This study |
| Anqing | Lower | 2009.6~9 | 0.51~1.1 | 14 | 1.0~2.4 | 4.01~7.64 |  | Wang et al., 2015 [78] |
| Datong | Lower | 2009.6~9 | 0.56~1.34 | 15 | 0.8~2.8 | 2.73~8.68 |  |  |
| Maanshan | Lower | 2009.6~9 | 0.55~1.27 | 15 | 1.1~2.5 | 4.23~7.39 |  |  |
| Nanjing | Lower | 2006~2015 | 0.51~1.61 | 16.53~24.21 | 1.28~4.80 | 1.30~12.41 | 5.59 | This study |
| Anqing | Lower | 2009.1~12 | 0.83 | 13.8 | 1.79 |  | 6.02 | Yan et al., 2012 [54] |
| Datong | Lower | 2009.1~12 | 0.95 | 15.6 | 1.51 |  | 5.33 |  |
| Maanshan | Lower | 2009.1~12 | 0.95 | 15.5 | 1.93 |  | 6.40 |  |

**refers to the representative sites of *k*_600_ in the Yangtze River.

The calculation equation is *k*_600_=1.0+1.719w^0.5^h^-0.5^+2.58u_10_.

# References

50. Chen J, Wu X and Finlayson BL *et al*. Variability and trend in the hydrology of the Yangtze River, China: annual precipitation and runoff. *J Hydrol* 2014; **513**: 403-412.

51. Zhang L, Xue M and Wang M *et al*. The spatiotemporal distribution of dissolved inorganic and organic carbon in the main stem of the Changjiang (Yangtze) River and the effect of the Three Gorges Reservoir. *J Geophys Res-Biogeosciences* 2014; **119**: 741-757.

52. Beaulieu J, Shuster W and Rebholz J. Nitrous oxide emissions from a large, impounded river: The Ohio River. *Environ Sci Technol* 2010; **44**: 7527-7533.

53. Zhao J, Zhang G and Wu Y *et al*. Distribution and emission of nitrous oxide from the Changjiang River. *Acta Scien Circum* 2009; **29**: 1995-2002.

54. Yan W, Yang L and Wang F *et al*. Riverine N_2_O concentrations, exports to estuary and emissions to atmosphere from the Changjiang River in response to increasing nitrogen loads. *Global Biogeochem Cycles* 2012; **26**.

55. Lewis E, Wallace D and Allison LJ. Program developed for CO_2_ system calculations. Brookhaven National Lab., Dept. of Applied Science, Upton, NY (United States); Oak Ridge National Lab, Carbon Dioxide Information Analysis Center, TN (United States), 1998.

56. Ran L, Lu, XX and Richey JE *et al*. Long-term spatial and temporal variation of CO_2_ partial pressure in the Yellow River, China. *Biogeosciences* 2015; **12**: 921-932.

57. Yao G, Gao Q and Wang Z *et al*. Dynamics of CO_2_ partial pressure and CO_2_ outgassing in the lower reaches of the Xijiang River, a subtropical monsoon river in China. *Sci Total Environ.* 2007; **376**: 255-266.

58. Li S, Lu X and Bush RT. CO_2_ partial pressure and CO_2_ emission in the Lower Mekong River. *J Hydrol* 2013; **504**: 40-56.

59. Cole JJ, Caraco NF and Kling GW *et al*. Carbon Dioxide Supersaturation in the Surface Waters of Lakes. *Science* 1994; **265**, 1568-1570.

60. Raymond PA, Hartmann J and Lauerwald R *et al*. Global carbon dioxide emissions from inland waters. *Nature* 2013; **503**: 355-359.

61. Ran L, Lu XX and Yang H *et al*. CO_2_ outgassing from the Yellow River network and its implications for riverine carbon cycle. *J Geophys Res-Biogeosci* 2015; **120**: 1334-1347.

62. Hunt CW, Salisbury JE and Vandemark D *et al*. Contribution of non-carbonate anions to total alkalinity and overestimation of *p*CO_2_ in New England and New Brunswick rivers. *Biogeosciences* 2011; **8**: 3069-3076.

63. Thauer RK and Shima S. Methane as fuel for anaerobic microorganisms. *Ann NY Acad Sci* 2008; **1125**: 158-170.

64. O'Connor FM, Boucher O and Gedney N *et al*. Possible role of wetlands, permafrost, and methane hydrates in the methane cycle under future climate change: A review. *Rev Geophys* 2010; **48**: G4005.

65. Bubier JL and Moore TR. An ecological perspective on methane emissions from northern wetlands. *Trend Ecol Evolut* 1994; **9**: 460-464.

66. Yvon-Durocher G, Montoya JM and Woodward G *et al*. Warming increases the proportion of primary production emitted as methane from freshwater mesocosms. *Global Change Biol* 2011; **17**: 1225-1234.

67. Yvon-Durocher G, Allen AP and Bastviken D *et al*. Methane fluxes show consistent temperature dependence across microbial to ecosystem scales. *Nature* 2014; **507**: 488.

68. Treat CC, Wollheim WM and Varner RK *et al*. Temperature and peat type control CO_2_ and CH_4_ production in Alaskan permafrost peats. *Global Change Biol* 2014; **20**: 2674-2686.

69. Bodelier PL and Laanbroek HJ. Nitrogen as a regulatory factor of methane oxidation in soils and sediments. *FEMS Microbiol Ecol* 2004; **47**: 265-277.

70. Liu L and Greaver TL. A review of nitrogen enrichment effects on three biogenic GHGs: the CO_2_ sink may be largely offset by stimulated N_2_O and CH_4_ emission. *Ecol Lett* 2009; **12**: 1103-1117.

71. Xu Z, Zheng X and Wang Y *et al*. Effects of elevated CO_2_ and N fertilization on CH_4_ emissions from paddy rice fields. *Global Biogeochem Cycle* 2004; **18**: GB3009.

72. Song C, Yang G and Liu D *et al*. Phosphorus availability as a primary constraint on methane emission from a freshwater wetland. *Atmos Environ* 2012; **59**: 202-206.

73. Zhu X, Zhuang Q and Qin Z *et al*. Estimating wetland methane emissions from the northern high latitudes from 1990 to 2009 using artificial neural networks. *Global Biogeochem Cycle* 2013; **27**: 592-604.

74. Klein CAM, Novoa RSA and Ogle S *et al*. N_2_O emissions from managed soils, and CO_2_ emissions from lime and urea application. *IPCC guidelines for National greenhouse gas inventories, prepared by the National greenhouse gas inventories programme* 2006; **4**: 1-54.

75. Baulch HM, Dillon PJ and Maranger R *et al*. Night and day: short‐term variaton in nitrogen chemistry and nitrous oxide emissions from streams. *Freshwater Biol* 2012; **57**: 509-525.

76. Chen J, Cao W and Cao D *et al*. Nitrogen loading and nitrous oxide emissions from a river with multiple hydroelectric reservoirs. *Bull Environ Contam Toxicol* 2015; **94**: 633-639.

77. Chen N, Wu J and Zhou X *et al*. Riverine N_2_O production, emissions and export from a region dominated by agriculture in Southeast Asia (Jiulong River). *Agric Ecosyst Environ* 2015; **208**: 37-47.

78. Wang J, Chen N and Yan W *et al*. Effect of dissolved oxygen and nitrogen on emission of N_2_O from rivers in China. *Atmos Environ* 2015; **103**: 347-356.

79. Hertz J, Krogh A and Palmer RG. *Introduction to the theory of neural computation*. Addison-Wesley/Addison Wesley Longman, 1991.

80. Haykin S. *Neural networks: a comprehensive foundation*. Prentice Hall PTR, 1994.

81. Sadeghi B. A BP-neural network predictor model for plastic injection molding process. *J Mater Process Technol* 2000; **103**: 411-416.

82. Yan W, Mayorga E and Li X *et al*. Increasing anthropogenic nitrogen inputs and riverine DIN exports from the Changjiang River basin under changing human pressures. *Global Biogeochem Cycle* 2010; **24**.

83. Zhang GL, Zhang J and Liu SM *et al*. Nitrous oxide in the Changjiang (Yangtze River) Estuary and its adjacent marine area: Riverine input, sediment release and atmospheric fluxes. *Biogeosciences* 2010; **7**: 3505-3516.

84. Clough TJ, Bertram JE and Sherlock RR *et al*. Comparison of measured and EF5‐r‐derived N_2_O fluxes from a spring‐fed river. *Global Change Biol* 2006; **12**: 352-363.

85. Beaulieu JJ, Tank JL and Hamilton SK *et al*. Nitrous oxide emission from denitrification in stream and river networks. *Proc Natl Acad Sci USA* 2010; **108**: 214-219.

86. Wanninkhof R. Relationship between wind speed and gas exchange over the ocean. *J Geophys Res-Oceans* 1992; **97**: 7373-7382.

87. Wanninkhof R. Relationship between wind speed and gas exchange over the ocean revisited. *Limnol. Oceanogr-Meth* 2014; **12**: 351-362.

88. Zappa CJ, McGillis WR and Raymond PA *et al*. Environmental turbulent mixing controls on air-water gas exchange in marine and aquatic systems. *Geophys Res Lett* 2007; **34**: L10601.

89. Hall RO and Madinger HL. Use of argon to measure gas exchange in turbulent mountain streams. *Biogeosciences* 2018; **15**: 3085-3092.

90. Ulseth AJ, Hall RO and Boix CM *et al*. Distinct air–water gas exchange regimes in low- and high-energy streams. *Nat Geosci* 2019; **12**: 259-263.

91. Marzolf ER, Mulholland PJ and Steinman AD. Improvements to the Diurnal Upstream–Downstream Dissolved Oxygen Change Technique for Determining Whole-Stream Metabolism in Small Streams. *Can J Fish Aquat Sci* 1994; **51**: 1591-1599.

92. Wanninkhof R, Doney SC and Bullister JL *et al*. Detecting anthropogenic CO_2_ changes in the interior Atlantic Ocean between 1989 and 2005. *J Geophys Res-Oceans* 2010; **115**: C11.

93. Chen Z, Li J and Shen H *et al*. Yangtze River of China: historical analysis of discharge variability and sediment flux. *Geomorphology* 2001; **41**: 77-91.

94. Borges AV, Vanderborght JP and Schiettecatte LS *et al*. Variability of the gas transfer velocity of CO2 in a macrotidal estuary (the Scheldt). *Estuaries* 2004; **27**: 593-603.

95. Raymond PA and Cole JJ. Gas exchange in rivers and estuaries: Choosing a gas transfer velocity. *Estuaries* 2001; **24**: 312-317.

96. Liss PS and Merlivat L. Air-Sea Gas Exchange Rates: Introduction and Synthesis. In: Buat-Ménard P (ed). The Role of Air-Sea Exchange in Geochemical Cycling. Springer Netherlands: Dordrecht 1986; 113-127.

97. Cole JJ and Caraco NF. Atmospheric exchange of carbon dioxide in a low‐wind oligotrophic lake measured by the addition of SF_6_. *Limnol Oceanogr* 1998; **43**: 647-656.

98. Raymond PA, Zappa CJ and Butman D *et al*. Scaling the gas transfer velocity and hydraulic geometry in streams and small rivers. *Limnology and Oceanography: Fluids and Environments* 2012; **2**: 41-53.

99. Butman D and Raymond PA. Significant efflux of carbon dioxide from streams and rivers in the United States. *Nat Geosci* 2011; **4**: 839-842.

100. Yan W, Laursen AE and Wang F *et al*. Measurement of denitrification in the Changjiang River. *Environ Chem* 2004; **1**: 95-98.

101. Zhang GL, Zhang J and Liu SM *et al*. Nitrous oxide in the Changjiang (Yangtze River) Estuary and its adjacent marine area: Riverine input, sediment release and atmospheric fluxes. *Biogeosciences* 2010; **7**: 3505-3516.

102. Qu B, Aho KS and Li C *et al*. Greenhouse gases emissions in rivers of the Tibetan Plateau. *Sci Rep* 2017; **7**: 16573.

103. Dean WE and Gorham E. Magnitude and significance of carbon burial in lakes, reservoirs, and peatlands. *Geology* 1998; **26**: 535-538.

104. Mendonca R, Müller RA and Clow DW *et al*. Organic carbon burial in global lakes and reservoirs. *Nat Commun* 2017; **8**: 1694.

105. Mendonca R, Kosten S and Sobek S *et al*. Hydroelectric carbon sequestration. *Nat Geosci* 2012; **5**: 838-840.

106. Chen JS, Guan WR and Xia XH *et al*. Evolution in water quality and its relation with environmental acidification in the upper and middle reaches of the Yangtze river (in Chinese). *Acta scientiae circumstantiae* 1998; **3**: 43-48.

107. Le JX and Wang DC. Hydrochemical characteristics of rivers in Chin (in Chinese). *Acta Geographica Sinica* 1963; **29**: 1-13.

108. Shen HT. *Material flux of the Changjiang Estuary (in Chinese)*. Beijing: China Ocean Press, pp 176, 2001.

109. Zhang L, Xue M and Wang M *et al*. The spatiotemporal distribution of dissolved inorganic and organic carbon in the main stem of the Changjiang (Yangtze) River and the effect of the Three Gorges Reservoir. *J Geophys Res-Biogeosci* 2014; **119**: 741-757.

110. Li J. The impacts of chemical weathering of carbonate rock by sulfuric acid on the cycling of dissolved inorganic carbon in Changjiang River water (in Chinese). *Geochimica* 2010; **39**: 305-313.

111. Bastviken D, Tranvik LJ and Downing JA *et al*. Enrich-Prast, Freshwater methane emissions offset the continental carbon sink. *Science* 2011; **331**: 50.

112. Barros N, Cole JJ and Tranvik LJ *et al*. Carbon emission from hydroelectric reservoirs linked to reservoir age and latitude. *Nat Geosci* 2011; **4**: 593-596.

113. Deemer BR, Harrison JA and Li S *et al*. Greenhouse Gas Emissions from Reservoir Water Surfaces: A New Global Synthesis. *Bioscience* 2016; **66**: 949-964.

114. Prairie YT, Alm J and Beaulieu J *et al*. Greenhouse Gas Emissions from Freshwater Reservoirs: What Does the Atmosphere See? *Ecosystems* 2018; **21**: 1058-1071.

115. IPCC. *Climate Change 2013: The Physical Science Basis. Contribution of Working Group I to the Fifth Assessment Report of the Intergovernmental Panel on Climate Change.* Cambridge University Press: Cambridge, United Kingdom and New York, NY, USA, 2013.

116. Tremblay A., Varfalvy L and Roehm C *et al*. Greenhouse Gas Emissions - Fluxes and Processes: Hydroelectric Reservoirs and Natural Environments. Springer Berlin Heidelberg: Berlin, Heidelberg, pp 637-659, 2005.

117. Tremblay A, Lambert M and Gagnon L. Do hydroelectric reservoirs emit greenhouse gases? *Environ Manage* 2004; **33**: S509-S517.

118. Linn DM. Effect of Water-Filled Pore Space on Carbon Dioxide and Nitrous Oxide Production in Tilled and Nontilled Soils. *Soil Sci Soc Am J* 1984; **48**: 1267-1260.

119. Vachon D, Lapierre JF and Giorgio PA. Seasonality of photochemical dissolved organic carbon mineralization and its relative contribution to pelagic CO_2_ production in northern lakes. *J Geophys Res-Biogeosci* 2016; **121**: 864-878.

120. Guérin F and Abril G. Significance of pelagic aerobic methane oxidation in the methane and carbon budget of a tropical reservoir. *J Geophys Res-Biogeosci* 2007; **112**, G3.

121. Bastviken D, Ejlertsson J and Tranvik L. Measurement of Methane Oxidation in Lakes:  A Comparison of Methods. *Environ Sci Technol* 2002; **36**: 3354-3361.

122. Li S, Wang F and Luo W *et al*. Carbon dioxide emissions from the Three Gorges Reservoir, China. *Acta Geochimica* 2017; **36**: 645-657.

123. Bastviken D, Persson L and Odham G *et al*. Degradation of dissolved organic matter in oxic and anoxic lake water. *Limnol Oceanogr* 2004; **49**: 109-116.

124. Bastviken D, Cole J and Pace M *et al*. Methane emissions from lakes: Dependence of lake characteristics, two regional assessments, and a global estimate. *Global Biogeochem Cycle* 2004; **18**: GB4009.

125. Prairie YT and Giorgio PA. A new pathway of freshwater methane emissions and the putative importance of microbubbles. *Inland Waters* 2013; **3**: 311-320.

126. Deshmukh C, Guérin F and Labat D *et al*. Low methane (CH_4_) emissions downstream of a monomictic subtropical hydroelectric reservoir (Nam Theun 2, Lao PDR). *Biogeosciences* 2016; **13**: 1919-1932.

127. Yang Y, Liu CQ and Wu P *et al*. Study on methane emission of cascade reservoirs on Maotiaohe River Basin in summer (in Chinese). *Guizhou Water Power* 2009; **23**: 12-16.

128. Li S, Zhang Q and Bush RT *et al*. Methane and CO_2_ emissions from China’s hydroelectric reservoirs: a new quantitative synthesis. *Environ Sci Pollut Res* 2015; **22**: 5325-5339.

129. Guerin F, Abril G and Richard S *et al*. Methane and carbon dioxide emissions from tropical reservoirs: significance of downstream rivers. *Geophys Res Lett* 2006; **33**: L21407.

130. Wuebbles DJ and Hayhoe K.,Atmospheric methane and global change. *Earth-Sci Rev* 2002; **57**: 177-210.

131. Chen H, Yuan X and Chen Z *et al*. Methane emissions from the surface of the Three Gorges Reservoir. *J Geophys Res-Atmos* 2011; **116**: D21.

132. Yang L, Lu F and Wang X *et al*. Surface methane emissions from different land use types during various water levels in three major drawdown areas of the Three Gorges Reservoir. *J Geophys Res-Atmos* 2012; **117**.

133. Zhu D, Chen H and Yuan X *et al*. Nitrous oxide emissions from the surface of the Three Gorges Reservoir. *Ecol Eng* 2013; **60**: 150-154.

134. Chen H, Wu Y and Yuan X *et al*. Methane emissions from newly created marshes in the drawdown area of the Three Gorges Reservoir. *J Geophys Res-Atmos* 2009; **114**: D18.

135. Phillips FA, Leuning R and Baigent R *et al*. Denmead OT. Nitrous oxide flux measurements from an intensively managed irrigated pasture using micrometeorological techniques. *Agric For Meteorol* 2007; **143**: 92-105.

136. Lai X, Yin D and Finlayson BL *et al*. Will river erosion below the Three Gorges Dam stop in the middle Yangtze? *J Hydrol* 2017; **554**: 24-31.

137. Yuan W, Yin D and Finlayson B *et al*. Assessing the potential for change in the middle Yangtze River channel following impoundment of the Three Gorges Dam. *Geomorphology* 2012; **147-148**: 27-34.

138. Liu R, Men C and Liu Y *et al*. Spatial distribution and pollution evaluation of heavy metals in Yangtze estuary sediment. *Mar Pollut Bull* 2016; **110**: 564-571.

139. Dai Z and Liu JT. Impacts of large dams on downstream fluvial sedimentation: An example of the Three Gorges Dam (TGD) on the Changjiang (Yangtze River). *J Hydrol* 2013; **480**: 10-18.

140. Liu T, Zhang AN and Wang J *et al*. Integrated biogeography of planktonic and sedimentary bacterial communities in the Yangtze River. *Microbiome* 2018; **6**: 16.

141. Grams PE, Schmidt JC and Topping DJ. The rate and pattern of bed incision and bank adjustment on the Colorado River in Glen Canyon downstream from Glen Canyon Dam, 1956–2000. *Geol Soc Amer Bull* 2007; **119**: 556-575.

142. Dai Z and Liu JT. Impacts of large dams on downstream fluvial sedimentation: an example of the Three Gorges Dam (TGD) on the Changjiang (Yangtze River). *J Hydrol* 2013; **480**: 10-18.

143. Grams PE and Schmidt JC. Equilibrium or indeterminate? Where sediment budgets fail: Sediment mass balance and adjustment of channel form, Green River downstream from Flaming Gorge Dam, Utah and Colorado. *Geomorphology* 2005; **71**: 156-181.

144. Asaeda T and Rashid MH. The impacts of sediment released from dams on downstream sediment bar vegetation. *J Hydrol* 2012; **430**: 25-38.

145. Japan’s first coordinated sediment flushing/coordinated sediment sluicing with full drawdown-A close look, http://www.japanriver.or.jp/EnglishDocument/DB/file/005%20Hokuriku%2013, 2003.

146. Fu K, He D and Lu X. Sedimentation in the Manwan reservoir in the Upper Mekong and its downstream impacts. *Quatern Int* 2008; **186**: 91-99.

147. Guérin F, Abril G and Tremblay A *et al*. Nitrous oxide emissions from tropical hydroelectric reservoirs. *Geophys Res Lett* 2008; **35**: L06404.

148. Manyari WV and de Carvalho Jr OA. Environmental considerations in energy planning for the Amazon region: Downstream effects of dams. *Energ Policy* 2007; **35**: 6526-6534.

149. Smith VB and Mohrig D. Geomorphic signature of a dammed Sandy River: The lower Trinity River downstream of Livingston Dam in Texas, USA. *Geomorphology* 2017; **297**: 122-136.

150. Ouyang W, Shan Y and Hao F *et al*. Accumulated impact assessment of river buffer zone after 30 years of dam disturbance in the Yellow River basin. *Stoch Environ Res Risk Assess* 2013; **27**: 1069-1079.

151. Guérin F, Abril G and Richard S *et al*. Methane and carbon dioxide emissions from tropical reservoirs: significance of downstream rivers. *Geophys Res Lett* 2006; **33**: L21407.

152. Shin YH and Julien PY. Effect of flow pulses on degradation downstream of Hapcheon Dam, South Korea. *J Hydraul Eng* 2010; **137**: 100-111.

153. Wang HW and Kuo WC. Geomorphic Responses to a Large Check‐Dam Removal on a Mountain River in Taiwan. *River Res Appl* 2016; **32**: 1094-1105.

154. Peters RJ, Liermann M and McHenry ML *et al*. Changes in streambed composition in salmonid spawning habitat of the Elwha River during dam removal. *J Am Water Resour Assoc* 2017; **53**: 871-885.

155. Cai WJ, Guo X and Chen CTA *et al*. A comparative overview of weathering intensity and HCO_3_^−^ flux in the world's major rivers with emphasis on the Changjiang, Huanghe, Zhujiang (Pearl) and Mississippi Rivers. *Cont Shelf Res* 2008; **28**: 1538-1549.

156. Striegl RG, Dornblaser MM and McDonald CP *et al*. Carbon dioxide and methane emissions from the Yukon River system. *Global Biogeochem Cycle* 2012; **26**: GB0E05.

157. Dubois KD, Lee D and Veizer J. Isotopic constraints on alkalinity, dissolved organic carbon, and atmospheric carbon dioxide fluxes in the Mississippi River. *J Geophys Res-Biogeosci* 2010; **115**: G02018.

158. Telmer K and Veizer J. Carbon fluxes, *p*CO_2_ and substrate weathering in a large northern river basin, Canada: carbon isotope perspectives. *Chem Geol* 1999; **159**: 61-86.

159. Richey JE, Melack JM and Aufdenkampe AK *et al*. Outgassing from Amazonian rivers and wetlands as a large tropical source of atmospheric CO_2_. *Nature* 2002; **416**: 617.

160. Aufdenkampe AK, Mayorga E and Raymond PA *et al*. Riverine coupling of biogeochemical cycles between land, oceans, and atmosphere. *Front Ecol Environ* 2011; **9**: 53-60.

161. Raymond PA, Hartmann J and Lauerwald R *et al*. Global carbon dioxide emissions from inland waters. *Nature* 2013; **503**: 355.

162. Lauerwald R, Laruelle GG and Hartmann J *et al*. Spatial patterns in CO_2_ evasion from the global river network. *Global Biogeochem Cycle* 2015; **29**: 534-554.

163. Butman D and Raymond PA. Significant efflux of carbon dioxide from streams and rivers in the United States. *Nat Geosci* 2011; **4**: 839.

164. Zhai W, Dai M and Guo X. Carbonate system and CO_2_ degassing fluxes in the inner estuary of Changjiang (Yangtze) River, China. *Mar Chem* 2007; **107**: 342-356.

165. Wang F, Wang Y and Zhang J *et al*. Human impact on the historical change of CO_2_ degassing flux in River Changjiang. *Geochem Trans* 2007; **8**: 7.

166. Chen CTA, Zhai W and Dai M. Riverine input and air–sea CO_2_ exchanges near the Changjiang (Yangtze River) Estuary: Status quo and implication on possible future changes in metabolic status. *Cont Shelf Res* 2008; **28**: 1476-1482.

167. De Angelis MA and Scranton MI. Fate of methane in the Hudson River and estuary. *Global Biogeochem Cycle* 1993; **7**: 509-523.

168. Middelburg JJ, Nieuwenhuize J and Iversen N *et al.* Methane distribution in European tidal estuaries. *Biogeochemistry* 2002; **59**: 95-119.

169. Bartlett KB, Crill PM and Bonassi JA *et al*. Methane flux from the Amazon River floodplain: Emissions during rising water. *J Geophys Res-Atmos* 1990; **95**: 16773-16788.

170. Yan W, Laursen AE and Wang F *et al*. Measurement of denitrification in the Changjiang River. *Environ Chem* 2004; **1**: 95-98.

171. Richey JE, Devol AH and Wofsy SC *et al*. Biogenic gases and the oxidation and reduction of carbon in Amazon River and floodplain waters. *Limnol Oceanogr* 1988; **33**: 551-561.

172. Rajkumar AN, Barnes J and Ramesh R *et al*. Methane and nitrous oxide fluxes in the polluted Adyar River and estuary, SE India. *Mar Pollut Bull* 2008; **56**: 2043-2051.

173. Cole JJ and Caraco NF. Emissions of nitrous oxide (N_2_O) from a tidal, freshwater river, the Hudson River, New York. *Environ Sci Technol* 2001; **35**: 991-996.

174. Laursen AE and Seitzinger SP. Diurnal patterns of denitrification, oxygen consumption and nitrous oxide production in rivers measured at the whole‐reach scale. *Freshwater Biol* 2004; **49**: 1448-1458.

175 Dong L, Nedwell D and Colbeck I *et al*. Nitrous oxide emission from some English and Welsh rivers and estuaries. *Water Air Soil Poll* 2005; **4**: 127-134.

176. Li S, Wang F and Luo W *et al*. Carbon dioxide emissions from the Three Gorges Reservoir, China. *Acta Geochimica* 2017; **36**: 645-657.

177. Yao X, Liu D and Yang Z *et al*. Preliminary studies on the mechanism of winter dinoflagellate bloom in Xiangxi Bay of the Three Gorges Reservoir (in Chinese). *Res Environ Sci* 2012; **25**: 645-651.

178. Zhang M. Preliminary study on cyanobacterial bloom in Xiangxi Bay, Three Gorges Reservoir (in Chinese). *Wetland Sci* 2009; **7**: 230-236.

179. Sheng Z, Dan S and Zhang K. Trophic status analysis of the upper stream and backwater area in typical tributaries, Three Gorges Reservoir (in Chinese). *J Lake Sci* 2010; **22**: 201-207.

180. Li Z, Bai L and Guo J *et al*. Comparative study on water-air CO_2_, CH_4_ flux in two tributaries in the Three Gorges Reservoir, China (in Chinese). *Huanjing kexue* 2013; **34**: 1008-1016.

181. Wang J, Yan W and Chen N *et al*. Variations of river N_2_O saturations and emission factors in relation to nitrogen levels in China (in Chinese). *J Agro⁃Environ Sci* 2012; **31**: 1576.

182. Garnier J, Billen G and Vilain G *et al*. Nitrous oxide (N_2_O) in the Seine river and basin: Observations and budgets. *Agric Ecosyst Environ* 2009; **133**: 223-233.

183. Clough TJ, Buckthought LE and Casciotti KL *et al*. Nitrous Oxide Dynamics in a Braided River System, New Zealand. *J Environ Qual* 2011; **40**: 1532-1541.

184. Holmén K and Liss P. Models for air-water gas transfer: an experimental investigation. *Tellus B* 1984; **36**: 92-100.

185. Reay DS, Smith KA and Edwards AC. Nitrous oxide emission from agricultural drainage waters. *Global Change Biol* 2003; **9**: 195-203.

186. Venkiteswaran JJ, Wassenaar LI and Schiff SL. Dynamics of dissolved oxygen isotopic ratios: a transient model to quantify primary production, community respiration, and air–water exchange in aquatic ecosystems. *Oecologia* 2007; **153**: 385-398.

187. Rosamond MS, Thuss SJ and Schiff SL. Dependence of riverine nitrous oxide emissions on dissolved oxygen levels. *Nat Geosci* 2012; **5**: 715-718.

188. Baulch HM, Dillon PJ and Maranger R *et al*., Night and day: short-term variation in nitrogen chemistry and nitrous oxide emissions from streams. *Freshwater Biol* 2012; **57**: 509-525.

189. Venkiteswaran JJ, Schiff SL and Wallin MB. Large Carbon Dioxide Fluxes from Headwater Boreal and Sub-Boreal Streams. *Plos One* 2014; **9**: e101756.
